# Strength and Durability of Natural Fine-Grained Soil Stabilized with Fly Ash–Based Geopolymer: Effects of Sulfate Attack and Freeze–Thaw Cycles

**DOI:** 10.3390/ma19173750

**Published:** 2026-09-03

**Authors:** Firdevs Uysal

**Affiliations:** Department of Civil Engineering, Niğde Ömer Halisdemir University, 51240 Niğde, Türkiye; firdevsuysal@ohu.edu.tr

**Keywords:** soil stabilization, fly ash geopolymer, durability, sulfate resistance, freeze-thaw, microstructural analysis

## Abstract

Problematic fine-grained soils exhibit low strength and inadequate durability, highlighting the need for sustainable stabilization using eco-friendly binders. This study examined the strength development and durability of a natural CH soil (NSs) stabilized with fly ash (FA) based geopolymer exposed to sulfate attack and freeze–thaw (F–T) cycles. The effects of FA content (0–40%) and NaOH molarity (0–10 M) on unconfined compressive strength (UCS) were evaluated after 1, 7, 28 and 56 days of curing. Durability was assessed separately under accelerated laboratory conditions after 1, 3, 5, 7 and 11 F–T cycles and 7, 28 and 56 days of sulfate exposure. In non-activated specimens, FA contents of up to 30% enhanced the UCS primarily through the microfiller effect and possible time-dependent pozzolanic reactions. Alkali activation promoted the development of a compact binding matrix through the dissolution and polycondensation of aluminosilicate precursors, with the microstructural and chemical observations being consistent with the possible formation of C-(A)-S-H and/or N-A-S-H-type reaction products. F30M8 exhibited the highest strength, reaching a 56-day UCS of 1488.58 kPa compared with 282.46 kPa for untreated NSs. F30M8 retained approximately 94% of its UCS after 11 F–T cycles and 92% after 56 days of sulfate exposure. XRD, FTIR, and SEM-EDX analyses provided evidence of aluminosilicate restructuring and the development of a dense microstructure under alkaline activation. This refined matrix may have contributed to limiting sulfate- and ice-crystal-induced deterioration, thereby helping to preserve the structural integrity of the FA-based geopolymer-stabilized NS specimens, whereas untreated and non-activated FA-stabilized specimens disintegrated under sulfate exposure. These findings indicate that FA-based geopolymer stabilization has considerable potential for natural CH soil under the laboratory exposure conditions investigated in this study.

## 1. Introduction

Fine-grained soils often exhibit deficiencies in fundamental engineering properties, such as load-bearing capacity and excessive settlement behavior [1]. To overcome these inherent limitations, soil stabilization is adopted as an improvement technique. In this method, chemical components are incorporated into the soil and react with soil particles to form binding or cementitious structures that meet specific engineering requirements [2,3]. Within stabilization systems, binders play a critical role in enhancing structural integrity and water resistance by forming interparticle bonds [4]. However, most binders are unable to simultaneously achieve complete impermeability while maintaining mechanical performance and durability. Despite this limitation, the use of binders effectively reduces water absorption, allowing the soil to sustain a lower equilibrium moisture content [5]. A wide variety of binders, including Portland cement, lime, gypsum, FA, slag, nano-chemical additives, sodium bentonite, and calcium chloride, have been used to improve soil properties [6,7]. Portland cement and lime are among the most employed soil stabilization agents owing to their cost-effectiveness, well-documented engineering performance, and availability [8]. Despite these advantages, the manufacturing of these traditional binders requires a significant amount of energy and contributes to approximately 7–10% of global human-caused CO_2_ emissions [9,10]. Furthermore, cement-stabilized soils frequently encounter durability problems in harsh environments, particularly under sulfate attack and F–T cycles [11]. Under these conditions, the development of expansive compounds, notably ettringite and gypsum, generates stress within the soil and promotes cracking, mass loss, and progressive degradation of the soil matrix [12,13]. From an energy and environmental impact perspective, the environmental burdens associated with conventional stabilizers have intensified the need to develop and implement environmentally friendly binder alternatives for fine-grained soil stabilization [14]. In response to these environmental challenges, geopolymers, often referred to as green cements, have gained growing interest as eco-friendly alternatives to conventional binders [7,15,16]. These materials can utilize industrial solid wastes and exhibit low energy demand, low thermal conductivity, and enhanced resistance to acid attack [17]. Consequently, they are considered promising low-carbon alternatives to Portland cement, with research indicating approximately 60–80% lower CO_2_ emissions and lower energy consumption [16,18].

Geopolymer-based binders are increasingly recognized as sustainable options for improving fine-grained soils, owing to their potential to reduce environmental impacts while maintaining adequate engineering performance [14,19]. Zhang et al. [20] indicated the potential of geopolymer binders as alternative soil stabilizers and reported that these materials could provide higher compressive strength than traditional cement-based stabilization methods. Subsequent research by Phummiphan et al. [21] investigated the geopolymer stabilization of marginal lateritic soils using a binder produced from high-calcium FA. They attributed the observed strength enhancement to the development of calcium silicate hydrate (C-S-H) together with geopolymeric products. Hamed and Demiröz [22] applied the Taguchi approach to optimize the FA content and slag for geopolymer stabilization of high-plasticity clay (CH) soil and reported that the molarity of NaOH had a major influence on strength development. However, Abdullah et al. [23] indicated that the high activity index and buffering capacity of natural CH soil may hinder the effective mixing of the activator within the soil matrix, resulting in less uniformity compared to that of low-plasticity clay (CL). Recent findings by Lou et al. [24] emphasized the effect of clay mineralogy, showing that montmorillonite-rich soils tend to have higher reactivity but are more vulnerable to moisture-induced binder degradation than illite-rich matrices. Sahoo and Singh [25] explored the performance of expansive soils improved with slag-derived geopolymer binders under F–T cycling and compared their performance with soils stabilized with lime and Portland cement. Their findings were primarily applicable to slag-based systems, in which calcium-rich C-A-S-H serves as the principal stabilization product.

The available literature on soil alkali activation has largely focused on idealized soil models, such as pure kaolin and synthetic clays, thereby neglecting the complex mineralogical composition and structural heterogeneity inherent to natural soils [24]. In addition, a considerable proportion of the existing literature has emphasized CL soils, leaving the alkaline activation behavior of natural CH soils largely unexplored [23]. This research gap is critical because natural CH soils, characterized by high activity indices and a substantial capacity to absorb hydroxyl ions, can interfere with the pH-controlled dissolution of aluminosilicate source materials and impede the establishment of a homogeneous geopolymeric matrix [23]. Moreover, while durability against F–T cycles is a major concern for infrastructure in cold regions, studies on expansive soils remain limited and frequently focus on slag-rich systems where stabilization is primarily driven by calcium-based C-A-S-H gel [25,26]. Consequently, the progressive evolution of the microstructure under distinct environmental stressors, specifically up to 11 F–T cycles and extended sulfate exposure of up to 56 days, has not yet been systematically evaluated in FA-based geopolymer systems. To address these research gaps, this study comprehensively evaluates a natural CH soil stabilized with alkali-activated Class F FA. The study investigated the transition from a physical microfiller mechanism in non-activated mixtures to chemical bonding in geopolymerized systems. The microstructural and mineralogical findings were consistent with the possible formation of C-(A)-S-H and/or N-A-S-H-type reaction products under alkaline activation. By integrating UCS testing with XRD, FTIR, and SEM-EDX analyses, this study evaluates how alkali-activation-induced microstructural and mineralogical changes contribute to strength development and resistance to sulfate attack and F–T-induced deterioration. These results offer valuable experimental evidence regarding the sustainable stabilization of clayey soils exposed separately to F–T cycling and sulfate exposure.

## 2. Materials and Methods

### 2.1. Materials

**Figure 1:**
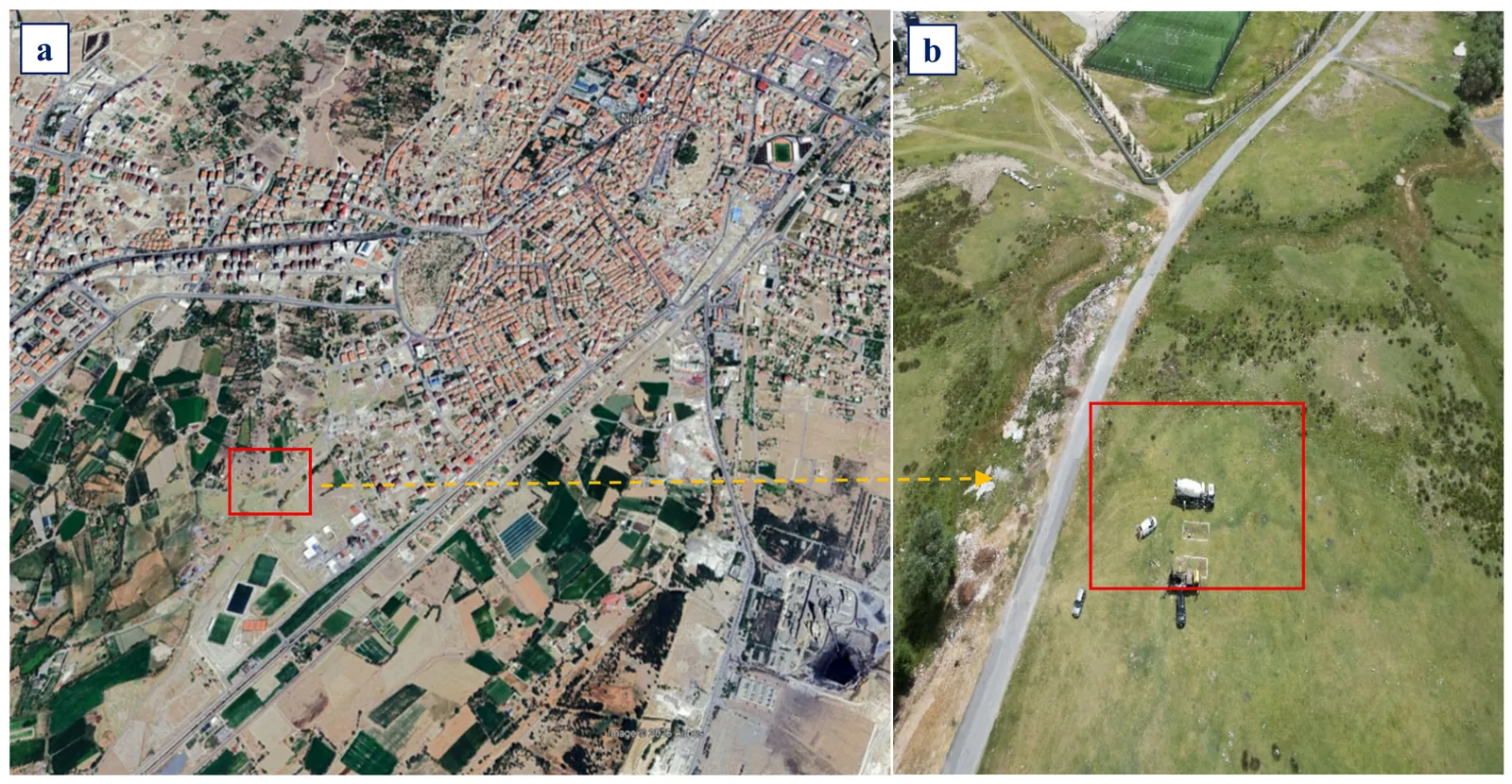
(**a**) Sampling location in Niğde, Türkiye; (**b**) field view of the site.

**Figure 2:**
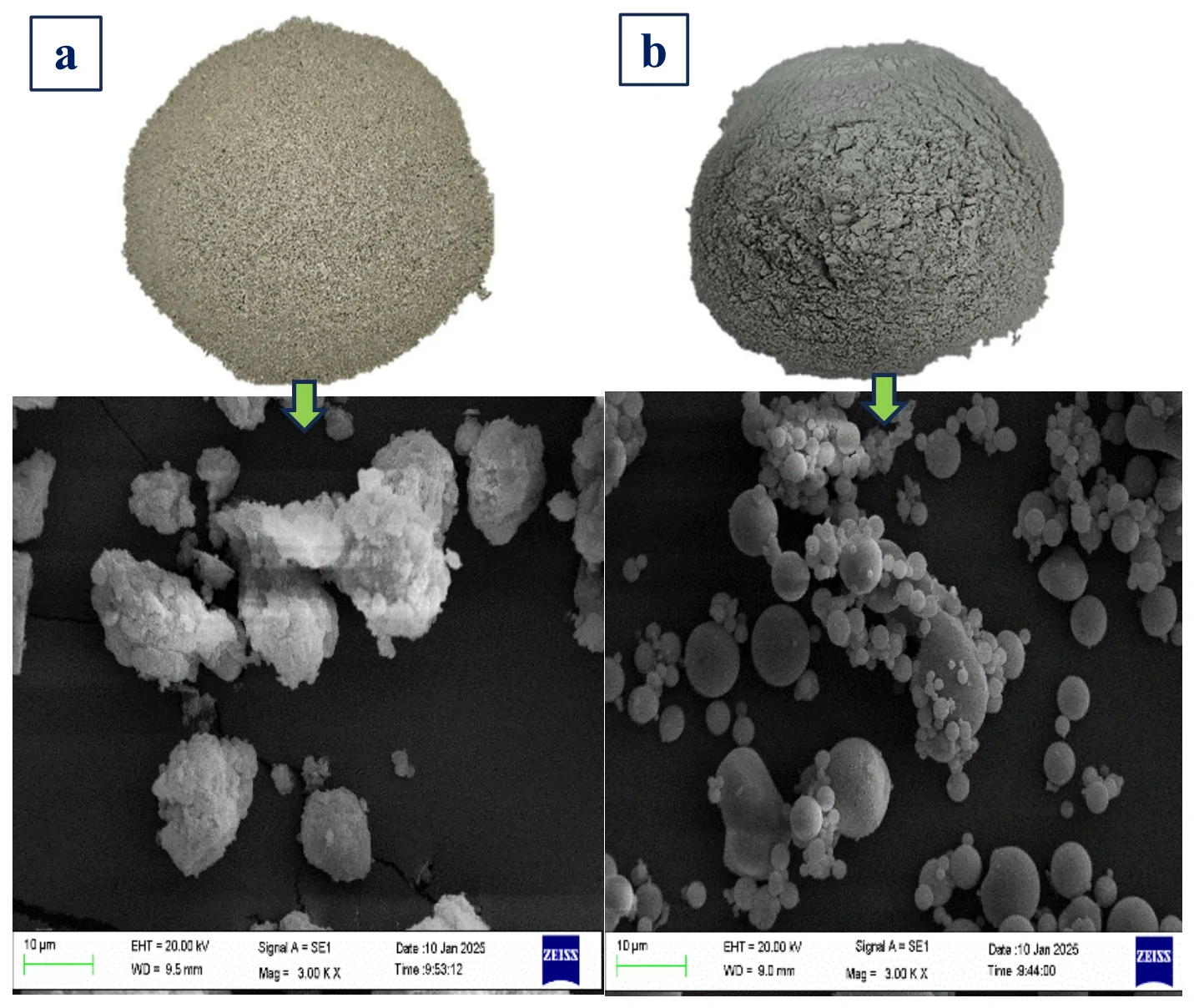
SEM images of the materials: (**a**) NS, (**b**) FA.

**Figure 3:**
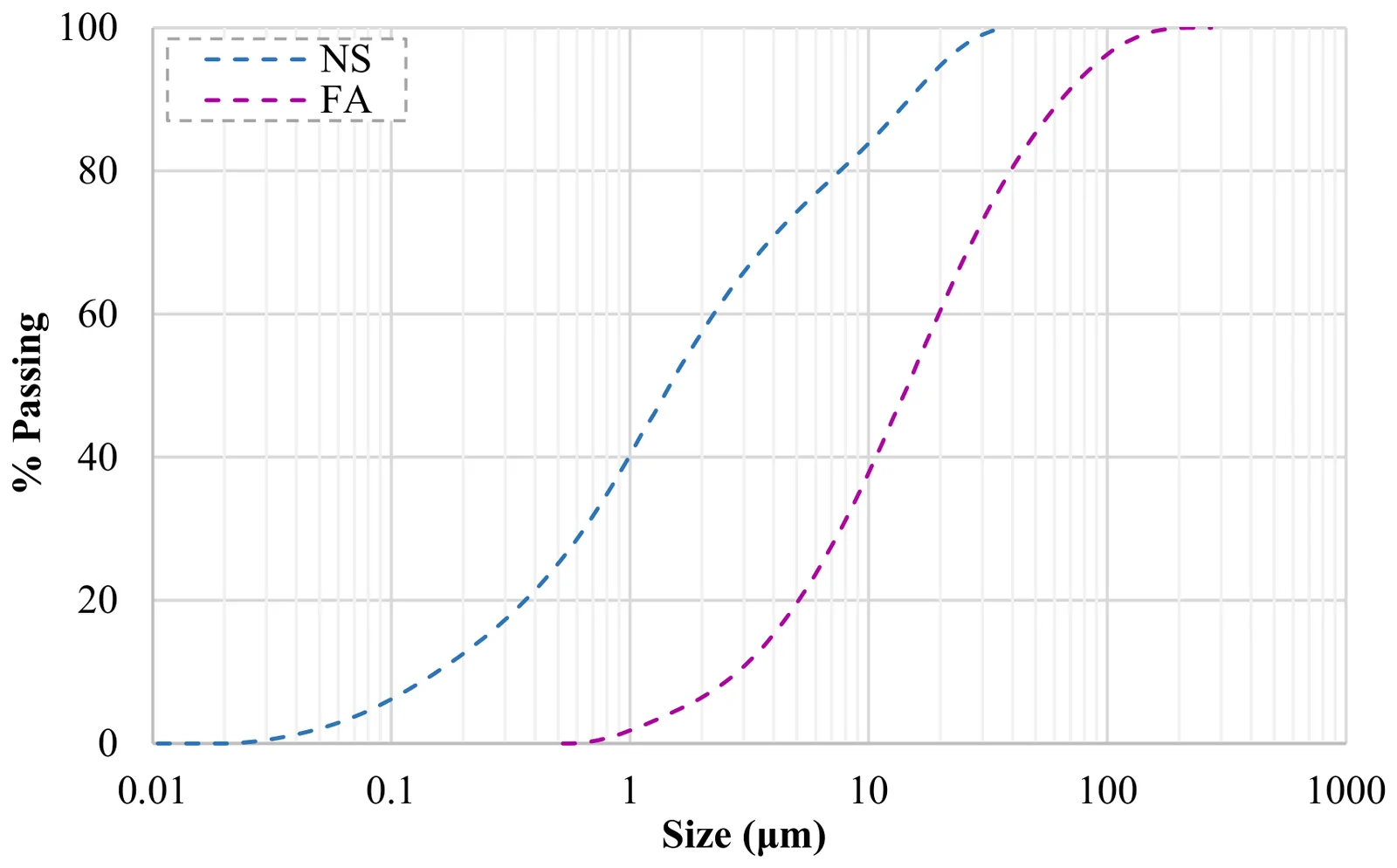
Particle-size distribution of NS and FA.

**Figure 4:**
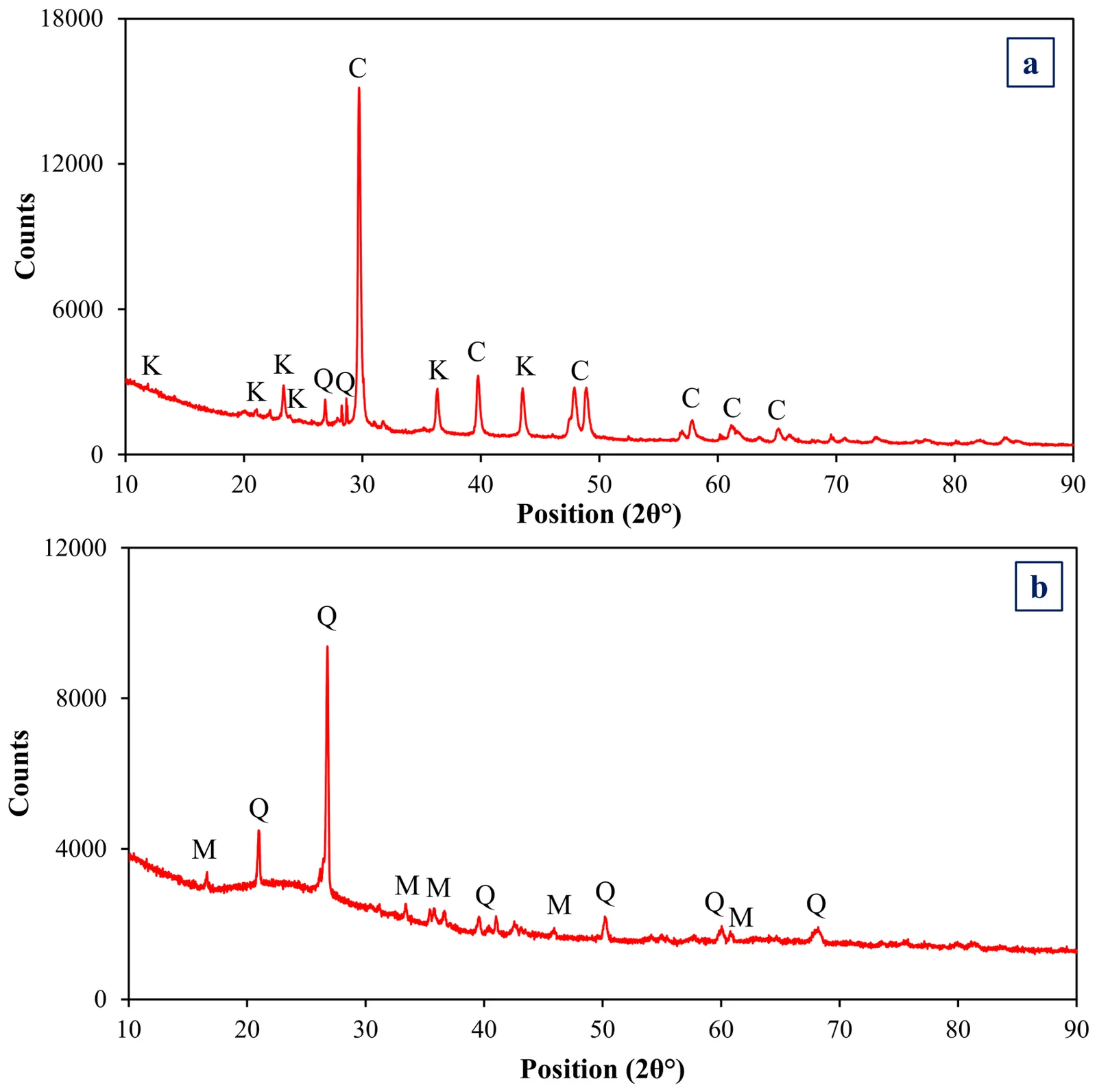
XRD patterns of NS (**a**), FA (**b**). C, calcite; K, kaolinite; Q, quartz; M, mullite.

The natural soil (NS) examined herein was sourced from a field in Niğde Province, Central Anatolia, Türkiye. The sampling location is situated at approximately 37°57′5.57″ N latitude and 34°39′35.63″ E longitude. Figure 1 presents the sampling site in Niğde and a field view of the study area. NS was classified as CH according to USCS. In this study, fly ash (FA) was used as the main aluminosilicate source for geopolymer stabilization. FA was obtained from Ekton Construction (Adana, Türkiye). FA demonstrated a specific gravity of 2.35 and a specific surface area of 5.353 m^2^/g, as determined by the multi-point BET method, which reflects a relatively large reactive surface area. This enables faster and more effective dissolution under alkaline activation, which may favor the development of a denser geopolymeric framework and consequently increase strength. The microstructures of the NS and FA were characterized using SEM (Figure 2). Table 1 summarizes the geotechnical properties of NS. XRF analysis was used to determine the chemical compositions of NS and FA, and the resulting data are summarized in Table 2. The particle-size distribution curves of NS and FA are provided in Figure 3, whereas their mineralogical assemblages, identified through XRD, are presented in Figure 4.

The XRD pattern of the NS displayed characteristic reflections of calcite (C), kaolinite (K), and quartz (Q), confirming the presence of common natural soil-forming minerals, which is consistent with the mineralogical profiles reported for various natural clayey soils in previous studies [27] (Figure 4a). Figure 4b shows that quartz (Q) is the primary crystalline phase in FA, accompanied by mullite (M). Q is considered an inert filler that exhibits limited reactivity during geopolymerization and contributes to the mechanical skeleton [28]. The presence of M, which is known for its excellent thermal and chemical stability at high temperatures, contributes to improved structural stability [29]. Sodium hydroxide (NaOH) with a purity of 98% in pellet form was procured from a local supplier. The density of the solid NaOH was 2.13 g/cm^3^.

**Table 1:** Index and compaction characteristics of the NS.

Parameter	Measured Value	Testing Standard
Specific gravity, G_S_	2.46	ASTM D854-23 [30]
Specific surface area, m^2^/g	36.428	ASTM D3663-20 [31]
Liquid limit, LL (%)	68.43	BS 1377-2:2022 [32]
Plastic limit, PL (%)	30.77	ASTM D4318-17e1 [33]
Plasticity index, PI (%)	37.66	ASTM D4318-17e1 [33]
Maximum dry density, MDD (kN/m^3^)	12.45	ASTM D698-12(2021) [34]
Optimum moisture content, OMC (%)	35	ASTM D698-12(2021) [34]

**Table 2:** Oxide compositions of the NS and FA.

Oxide Content (%)	NS	FA
SiO_2_	12.314	56.237
Al_2_O_3_	1.720	22.425
Fe_2_O_3_	2.066	10.247
CaO	44.628	2.138
MgO	2.180	0.670
K_2_O	0.641	2.666
TiO_2_	0.215	1.504
SO_3_	1.150	0.776
MnO	0.089	0.093
Na_2_O	0.432	1.320
ZnO	-	0.033
V_2_O_5_	-	0.081
BaO	0.023	0.165
P_2_O_5_	0.180	0.216
SrO	0.151	0.059
Cl	0.195	0.013
CeO_2_	-	0.161
ZrO_2_	-	0.048
NiO	0.011	0.043
CuO	-	0.015
LOI *	34.005	1.090

* LOI: Loss on ignition.

### 2.2. Mixture Design and Specimen Preparation

An extensive mixture design consisting of 21 different mixtures, including the control specimens (unstabilized NSs and NSs stabilized with FA), was implemented to assess the performance of NSs stabilized with FA-based geopolymer, as shown in Table 3. This procedure involved substituting FA for NS at dry-weight percentages of 0%, 10%, 20%, 30%, and 40%. NaOH molarity varied at 0, 4, 6, 8, and 10 M to assess its effects on geopolymerization and the stabilization performance of the specimens. The control specimens and specimens of NSs stabilized with FA-based geopolymer were designated based on their FA content and NaOH concentration as shown in Table 3. The NaOH solution was prepared before specimen preparation and left to reach ambient temperature to maintain chemical stability, because dissolving the NaOH pellets released heat. To ensure the reproducibility of the specimen preparation procedure, the mass-based quantities of NS, FA, sodium hydroxide pellets, and mixing water used in each mixture are presented in Table 4. For each mixture listed in Table 4, the combined dry mass of the natural soil and FA was fixed at 400 g. This quantity was selected based on preliminary trials, which showed that it was sufficient to completely fill the specimen mold during compaction while providing a small excess to facilitate uniform mixing and specimen preparation. In addition to the NS, the OMC and MDD values were determined separately for mixtures containing 10%, 20%, 30% and 40% FA. The corresponding OMC value for each FA content was used to determine the total mixing-water requirement of the relevant mixtures (Table 4).

**Figure 5:**
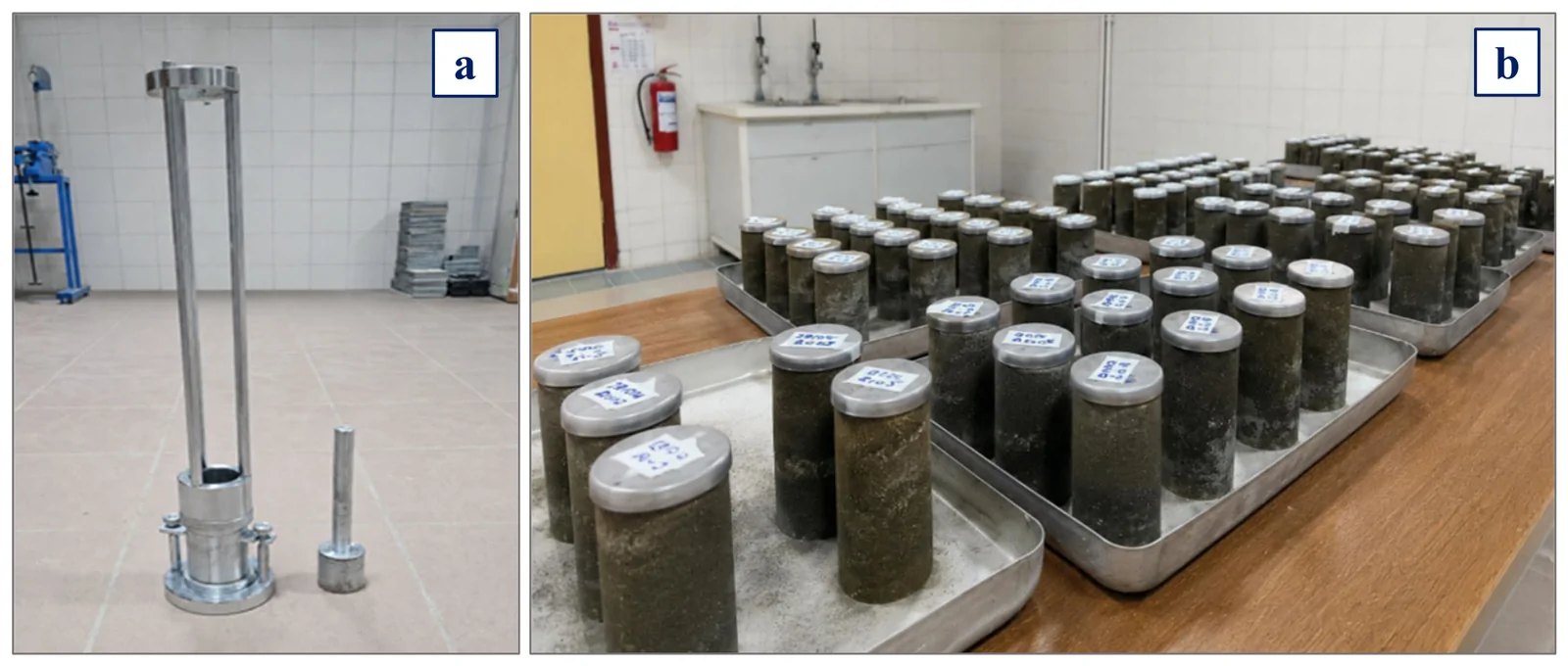
Cylindrical mold (**a**) and specimens of NSs stabilized with FA-based geopolymer (**b**).

The amount of water used to prepare the alkaline activating solution for each mixture was determined based on the corresponding OMC value (Table 4). During specimen preparation, dry NS was first blended with FA in a mixer to achieve a uniform dispersion of the precursor materials. The alkaline activating solution was subsequently introduced in increments, and blending proceeded until a uniformly stabilized mixture was obtained. To apply a compaction energy per unit volume corresponding to approximately 95% of the Standard Proctor effort of 592.5 kJ/m^3^ specified in ASTM D698-12(2021) [34], the mixtures were dynamically compacted in 50 mm × 100 mm cylindrical molds (diameter × height) in three equal layers [35]. Energy scaling calculations were performed based on the small mold volume (V = 196.35 cm^3^). Each of the three layers was compacted dynamically by applying 10 blows of a 1.5 kg mini hammer dropped from a controlled height of 25 cm, delivering a scaled compaction energy of 561.9 kJ/m^3^ (representing approximately 94.8% of the standard Proctor effort). The photograph of the specialized mini-mold and hammer setup used in this study is presented in Figure 5. The specimens were compacted using a target degree of compaction of 95% relative to the MDD determined for the corresponding FA replacement level. To prevent any premature geopolymerization and subsequent disruption of the consolidated binder matrix, the entire wet mixing and dynamic compaction process was strictly completed within 30 min of activator introduction. Three independent replicate specimens (*n* = 3) were prepared and tested for each mixture proportion, curing age, F–T cycle, and sulfate exposure period to ensure statistical reproducibility in this study.

The specimens of NSs stabilized with FA-based geopolymer were subsequently exposed to different curing periods and separate laboratory exposure conditions comprising F–T cycles and sulfate attack, as outlined in Table 3.

### 2.3. UCS Testing

The unconfined compressive strength (UCS) test was used to assess the strength development and durability of the control specimens and specimens of NSs stabilized with FA-based geopolymer after different curing days, F–T cycles, and sulfate attack. UCS measurements followed ASTM D2166-06 [36] at a constant displacement rate of 1.0 mm/min. All reported UCS and mass-change values represent the averages of three independent replicate measurements (*n* = 3).

### 2.4. Curing, Freeze–Thaw and Sulfate Attack Procedures

**Figure 6:**
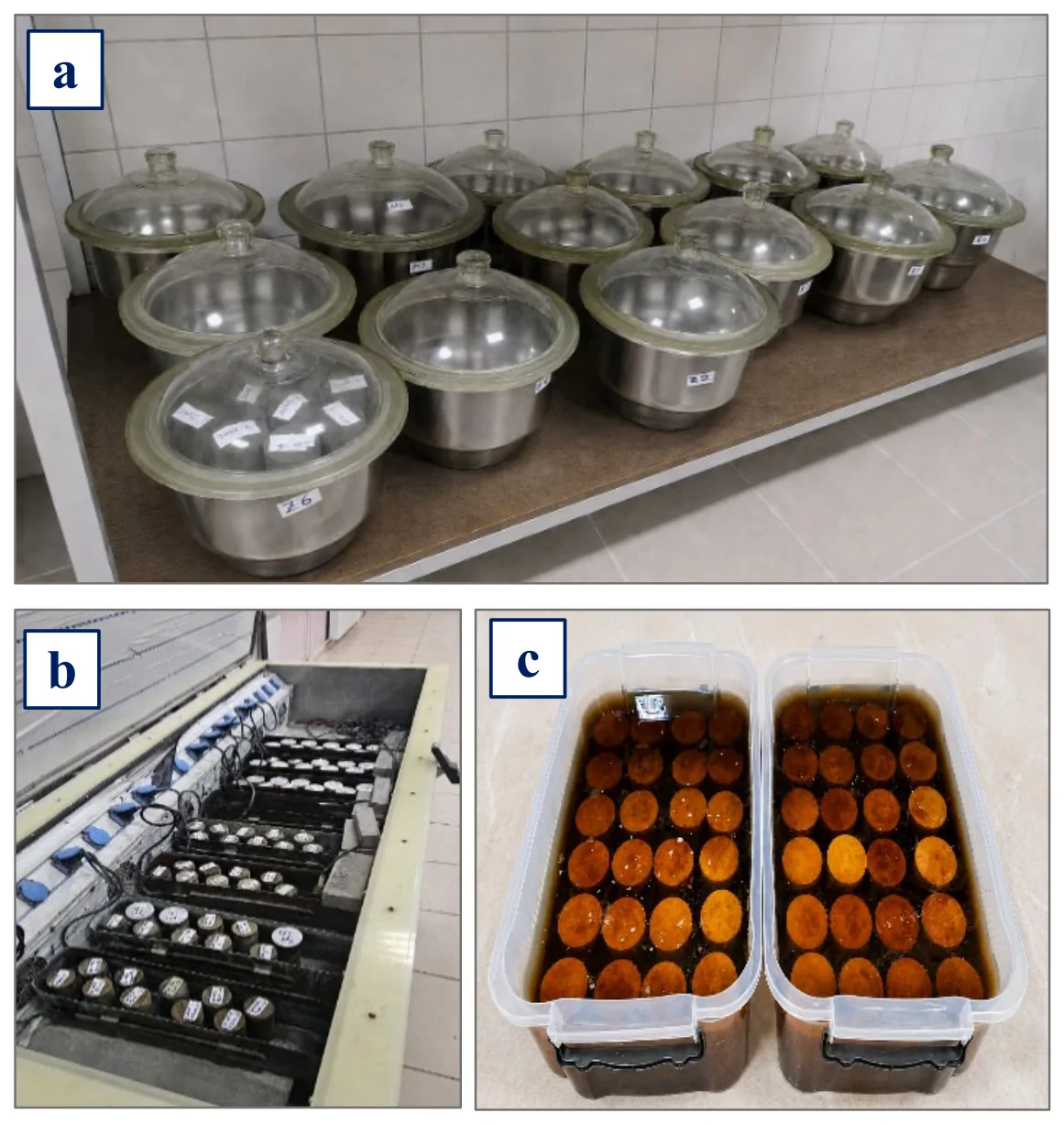
Durability conditions: (**a**) curing, (**b**) F–T, and (**c**) sulfate attack.

An extensive laboratory program was implemented to assess durability and environmental stability of NSs stabilized with FA-based geopolymer through a systematic series of protocols including curing, cyclic F–T exposure, and sulfate attack conditions (Figure 6). After compaction at the OMC and MDD, the soil specimens were placed in a sealed desiccator to avoid moisture evaporation and maintained at 23 ± 2 °C under regulated laboratory conditions (Figure 6a). The curing conditions were selected to promote alkali-activation reactions and facilitate the development of a stable soil matrix [37]. The curing process was conducted with two primary objectives: to provide a systematic assessment of the performance of NSs stabilized with FA-based geopolymer. First, the effect of curing time on the strength evolution of stabilized soils was examined. For this purpose, specimens were subjected to curing in controlled laboratory conditions for 1, 7, 28, and 56 days, followed by UCS measurement at each specified age. The 1-day curing period was used to evaluate early-age strength development, whereas the 7 and 28-day curing periods served as reference intervals commonly reported in the literature [14,38]. In addition, a 56-day curing period was included to assess the later-age strength development associated with continued time-dependent reactions. Second, a 28-day curing period was adopted prior to durability assessments to ensure sufficient geopolymerization and strength development before exposure to aggressive environmental conditions. Therefore, all specimens intended for F–T and sulfate resistance tests were cured for 28 days [39]. The UCS values obtained immediately after this initial curing period were used as the reference values for the durability assessments. Accordingly, the subsequent UCS changes are interpreted as the net responses of the specimens under the applied exposure conditions and may also include contributions from continued time-dependent reactions and, in the sulfate exposure tests, water-immersion effects.

The resistance of the NSs stabilized with FA-based geopolymer was assessed using closed-system cyclic F–T tests (Figure 6b). The specimens were subjected to repeated freezing and thawing after 28 days of curing. The testing procedure followed a modified protocol based on ASTM D560/D560M-16 [40]. Therefore, the procedure did not fully comply with all provisions of the standard. The cycle duration and temperatures, water-supply conditions, brushing procedure, and total number of cycles were modified to accommodate the dimensions and cohesive nature of the specimens and to permit subsequent UCS testing. At the Proctor OMC = 35% and MDD = 12.45 kN/m^3^, the theoretical initial degree of saturation of the NS was calculated as approximately 91.8%. This value represents the Proctor reference condition and should not be interpreted as a direct measurement for every test specimen. The freezing and expansion of pore water induce internal stresses that initiate microcracks, disrupt interparticle bonds and progressively increase the void and crack content within the soil matrix [41]. The specimens were examined after 1, 3, 5, 7 and 11 cycles to assess the progression of structural degradation and retention of mechanical integrity. The number of cycles was selected by considering previous studies that used 10–12 cycles to evaluate the F–T resistance of stabilized soils and geopolymer-based materials [42,43]. Each F–T cycle consisted of freezing the specimens in closed metal containers at −20 ± 3 °C for 12 h, followed by thawing at +20 ± 2 °C for 12 h [26,44]. Separate water-filled containers were placed inside the chamber to minimize specimen desiccation without direct contact between the specimens and water. Following the designated F–T cycles, the specimen durability was quantified based on mass loss measurements and UCS tests, enabling a detailed assessment of resistance to F–T-induced deterioration. The wire-brushing procedure specified in ASTM D560/D560M-16 [40] was omitted to prevent mechanical abrasion. Therefore, the measured mass loss primarily reflected material that naturally spalled or detached from the specimen surfaces during F–T cycling, without any additional loss caused by the wire-brushing procedure. For mass determination, oven-drying to constant mass was not applied because the specimens were subsequently used for UCS testing. After each designated F–T cycle, the specimens were gently rinsed with a small amount of deionized water without rubbing or brushing. Excess surface water was then gently blotted using a damp, lint-free absorbent cloth. The specimens were weighed under saturated surface-dry (SSD) conditions within 2 min using an electronic balance with a precision of 0.01 g. The mass loss after F–T cycles was calculated using Equation (1):

(1) $$W_{loss}(\%)=\frac{M_{i}-M_{FT}}{M_{i}}\times 100$$

where *M_i_* is the initial SSD mass of the cured specimen before durability testing (g) and *M_FT_* is the SSD mass of the specimen after F–T cycles (g).

To assess sulfate resistance of the stabilized soil specimens under aggressive groundwater conditions, a sulfate attack protocol was implemented (Figure 6c). After curing for 28 days, the specimens were completely submerged in a sodium sulfate (Na_2_SO_4_) solution. A 2.5% Na_2_SO_4_ concentration (0.176 mol/L) was selected based on the sulfate exposure conditions adopted by Kamon et al. [45]. To provide a recognized reference for the severity of this exposure condition, the selected concentration was also compared with the sulfate exposure classes specified in ACI 318-19 [46]. The 2.5% Na_2_SO_4_ solution corresponds to an approximate dissolved sulfate-ion (SO_4_^2−^) concentration of 16,900 mg/L. This value exceeds the threshold of 10,000 mg/L specified for the S3 (very severe) sulfate exposure class in ACI 318-19. Although this classification was developed for concrete structures rather than stabilized soils, it provides an established contextual benchmark indicating that the adopted solution represents a very severe sulfate exposure condition. The specimens were submerged in Na_2_SO_4_ solution for 7, 28, and 56 days [47,48]. The sulfate solution was renewed at seven-day intervals to maintain a stable sulfate concentration throughout exposure.

Sulfate-induced degradation was characterized by mass change analysis and UCS testing. The specimens were prepared and weighed using the same SSD procedure, weighing time, and balance precision described above for the F–T tests. At each designated sulfate exposure period, the specimens were removed from the solution and gently rinsed with a small amount of deionized water to remove residual surface solution and loosely attached salts before SSD weighing. The mass change after sulfate exposure was calculated using Equation (2):

(2) $$∆M(\%)=\frac{M_{t}-M_{i}}{M_{i}}\times 100$$

where *M_i_* is the initial SSD mass of the specimen before sulfate immersion (g), and *M_t_* is its SSD mass after the designated sulfate exposure period (g). A positive Δ*M* value indicates a net mass gain, whereas a negative value indicates a net mass loss.

A specimen was classified as disintegrated when it lost its original cylindrical geometry and structural cohesion to an extent that prevented its removal, handling, weighing, or UCS testing as an intact specimen. For specimens meeting this criterion, sulfate exposure was terminated, and the subsequent UCS and mass-change measurements were recorded as unavailable durability-failure outcomes rather than being assigned a value of zero.

### 2.5. Microstructural Characterizations

Microstructural investigations were conducted to clarify the underlying processes controlling strength evolution and durability of NS specimens treated with FA-based geopolymers. SEM-EDX, XRD, and FTIR characterizations were performed using Zeiss Gemini SEM500-Element EDX: Ametek (Meerbusch, Germany; Berwyn, PA, USA), EMPYREAN Advance Diffractometer, and Vertex 70 FTIR spectroscopy (Empyrean, Munich, Germany), respectively. SEM analyses were performed at magnifications of 1.00 kx and 3.00 kx at an acceleration voltage of 20.00 kV, with working distances of 10, 10.5, 11.0, 11.5, 13, and 15 mm. Before SEM examination, residual moisture was removed by drying, after which the specimens received a thin gold coating to improve image quality. XRD patterns were obtained using a PANalytical/Empyrean diffractometer (Malvern Panalytical, Almelo, The Netherlands) equipped with a Cu anode and a high-resolution 256 × 256 pixel detector. The measurements were performed using Cu-Kα radiation (Kα_1_ = 1.540598 Å and Kα_2_ = 1.544426 Å), with the X-ray tube operated at 45 kV and 40 mA. Before analysis, the samples were finely ground and dehydrated at 50 °C. The specimens were scanned at a constant angular velocity of 3.863° min^−1^ in continuous-scan mode over a 2θ range of approximately 5–90°, with an effective data-point interval of approximately 0.013°. Background subtraction, profile fitting and phase identification were performed using PANalytical HighScore Plus software (version 4.0, 4.0.0.119037) coupled with the ICDD PDF-2 database. The crystalline phases were identified and validated using the following standard reference card numbers: Quartz (Q, PDF# 01-085-0795), Calcite (C, PDF# 01-072-1937), Kaolinite (K, PDF# 01-074-1785), Mullite (M, PDF# 01-079-1455), Gismondine (Gi, PDF# 00-020-0453), Gypsum (Gy, PDF# 01-070-0982), and Anorthite (A, PDF# 01-089-1459). For consistency and clarity, only the 2θ range of 10–90° is presented in this study. FTIR analyses were performed using the KBr pellet method across a spectral interval of 400–4000 cm^−1^.

### 2.6. Statistical Analysis

All UCS and durability tests were performed using three independently prepared replicate specimens for each mixture and test condition (*n* = 3). The test results are reported as mean ± standard deviation. A three-way analysis of variance (ANOVA) was performed on the UCS results of the stabilized soil specimens to evaluate the main and interaction effects of FA content (10%, 20%, 30%, 40%), NaOH concentration (0, 4, 6, 8, 10 M), and curing age (1, 7, 28, 56 days). The significance level was set at α = 0.05. Post-hoc comparisons were conducted using Tukey’s Honestly Significant Difference (HSD) test to identify statistically significant differences between individual mixture codes.

## 3. Results and Discussion

### 3.1. Strength Development of NSs Stabilized with FA-Based Geopolymer

**Figure 7:**
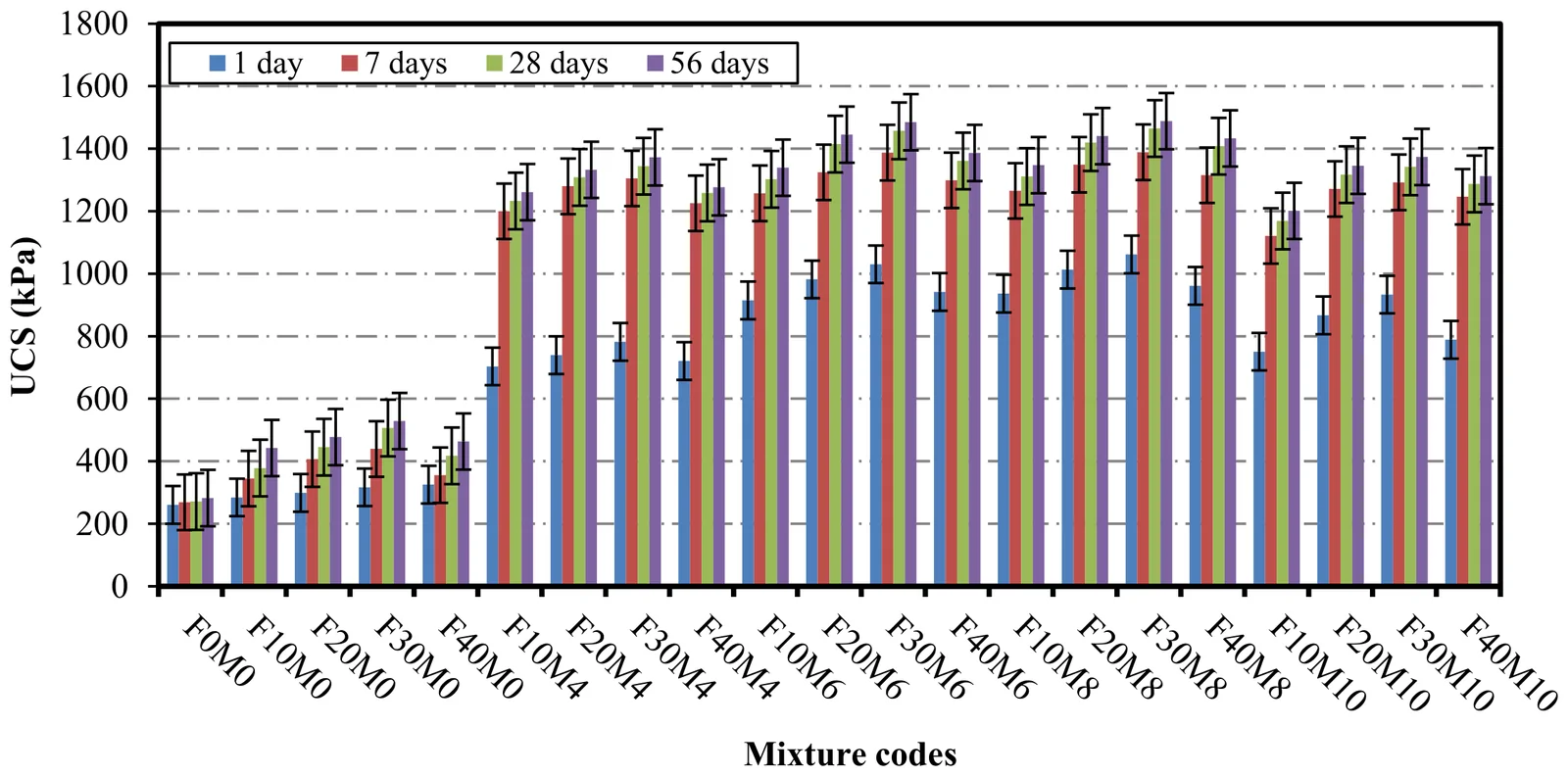
Effect of curing time on the UCS of all mixtures (Data are presented as mean values, and the error bars represent ± one standard deviation based on three independently prepared replicate specimens. This definition applies to all subsequent figures containing error bars).

The development of UCS for the unstabilized NSs (F0M0), NSs stabilized with FA, and NSs stabilized with FA-based geopolymer at curing ages of 1, 7, 28, and 56 days is illustrated in Figure 7. F0M0 recorded the minimum UCS values throughout the curing period among all mixtures. The NSs stabilized with FA exhibited a gradual increase in UCS as the FA dosage increased to 30%, then decreased when the FA dosage reached 40%. FA contributed to the enhancement of UCS only up to a 30% replacement level, primarily through the filler effect, whereby fine spherical FA particles filled interparticle voids within the soil skeleton, leading to matrix densification [49]. Raising the FA dosage to 40% caused a decline in UCS. This decline is mainly associated with the excessive presence of unreacted FA, which behaves as an inert inclusion, disrupting soil framework continuity [50]. In addition to the microfilling action of FA, the curing time contributed to the progressive improvement in the UCS of the NSs stabilized with FA (Figure 7). For example, the UCS of the F30M0 mixture increased from 316.82 kPa after 1 day to 439.57, 506.30, and 528.88 kPa at curing ages of 7, 28, and 56 days, respectively, indicating that the measured strength evolution cannot be attributed solely to the filler effect. Although XRF analysis indicated a high total calcium content expressed as CaO equivalent, XRD identified calcite as the principal calcium-bearing phase in the NS. Because soluble-calcium concentrations were not measured, the extent to which calcium from the NS participated in the reactions could not be directly determined. Therefore, the time-dependent development of F30M0 may reflect the combined contribution of the microfiller effect and possible pozzolanic reactions between FA-derived reactive silica and alumina and any calcium available under the curing conditions.

The NSs stabilized with the FA-based geopolymer exhibited substantially higher UCS values at all curing ages than the untreated NSs and NSs stabilized with FA (Figure 7). In the presence of NaOH, alkaline activation initiated geopolymerization by dissolving aluminosilicate source phases. The substantial increase in UCS observed after only one day of curing indicates the rapid formation of geopolymerization reaction products. For example, after one day of curing, the NaOH-activated F30M8 achieved a UCS of 1062.01 kPa, whereas the corresponding non-activated F30M0 exhibited a UCS of 316.82 kPa, representing an approximately 3.4-fold increase. This rapid strength gain highlights the efficiency of hydroxyl (OH^−^) ions in cleaving the resistant Si-O-Si and Al-O-Si bonds in FA, thus promoting the generation of geopolymeric reaction products. Strength increased most rapidly from 7 to 28 days, after which the rate of UCS gain progressively slowed.

Across all mixtures, the 28-day UCS values corresponded to approximately 85.48–98.59% of the respective 56-day values, with an overall average of 96.57%. For the alkali-activated mixtures, this ratio ranged from 97.20% to 98.59%, with an average of approximately 97.96%. These findings indicate that most of the measured strength development in the alkali-activated specimens had occurred by the end of the initial 28-day curing period. Accordingly, although continued time-dependent reactions during the subsequent exposure periods cannot be entirely excluded, their potential contribution to further strength development was likely limited. The increase in UCS was not proportional to the NaOH concentration. Raising the NaOH molarity from 4 M to 8 M produced a marked increase in the UCS of the NSs stabilized with FA. This trend agrees with previous studies, which showed that increasing the NaOH concentration enhanced aluminosilicate dissolution, which accelerated geopolymerization and increased compressive strength [39,51,52]. However, a further increase to 10 M resulted in a reduction in UCS, indicating that excessive alkalinity may adversely affect geopolymerization. This finding suggests that although increasing the NaOH concentration generally promotes geopolymerization and strength development, as reported by Chenna et al. [53], further increases in alkali concentration may not always result in additional strength enhancement. Bilondi et al. [54] also reported that an optimum NaOH concentration is essential for effective geopolymerization, whereas alkali concentrations lower and above the optimum level reduce the UCS of geopolymer-stabilized specimens. Among all mixtures, the F30M8 mixture was identified as the optimum mixture, achieving the maximum UCS of 1488.58 kPa at a curing age of 56 days. This performance suggests that an 8 M NaOH concentration provided sufficient alkalinity to enhance the dissolution of aluminosilicate source phases and facilitate the ensuing geopolymerization reactions. Shi et al. [55] found that specimens treated with 8 M NaOH achieved the maximum UCS, which was associated with cementitious product coating the clay particles, as confirmed by SEM and XRD analyses. Likewise, Turkane and Chouksey [56] reported that specimens activated with 8 M NaOH exhibited higher UCS than those activated with 6 M NaOH. They attributed this improvement to the enhanced dissolution of aluminosilicate species, greater geopolymer gel formation, and formation of a more compact matrix, as verified by SEM, EDX, XRD, and FTIR analyses.

### 3.2. Effect of Freeze–Thaw Cycles on Mechanical Performance

**Figure 8:**
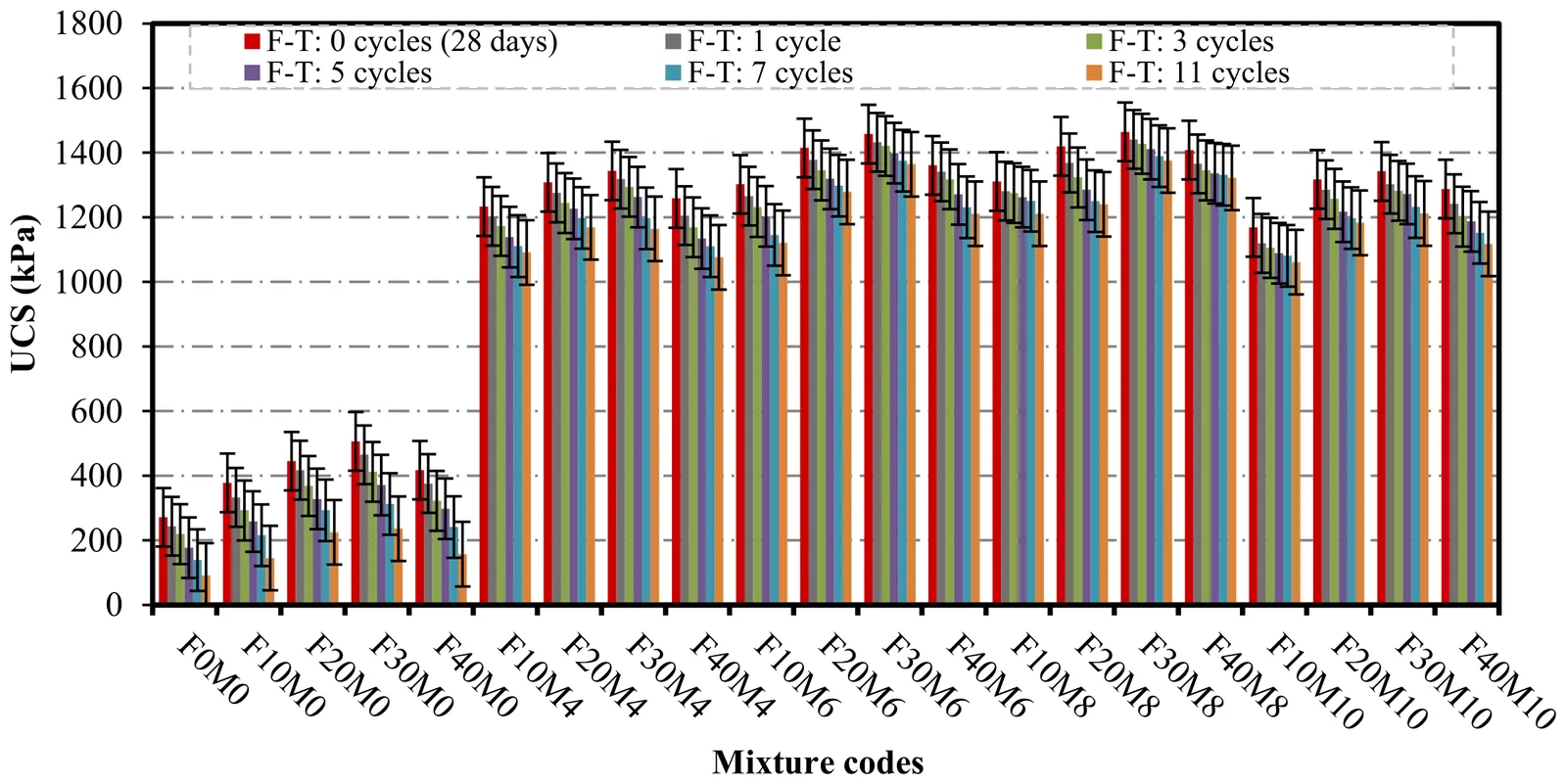
Effect of F–T cycles on the UCS of all mixtures.

The changes in UCS for F0M0, NSs stabilized with FA, and NSs stabilized with FA-based geopolymer after 1, 3, 5, 7, and 11 F–T cycles are presented in Figure 8. Regardless of the mixture composition, subjecting all specimens to repeated F–T cycles induced a progressive degradation in UCS from the first to the eleventh cycle. These changes were evaluated relative to the corresponding 28-day reference values and, because age-matched curing controls were not included, are interpreted as net responses under the applied F–T conditions rather than effects attributable exclusively to F–T action. The observed mechanical deterioration may be partly associated with pore-water freezing, which can generate crystallization pressure and hydrostatic stress within the soil matrix, potentially contributing to progressive structural damage [57,58,59]. This trend agrees with the findings of Li et al. [60], who reported that the rate of strength loss decreased after the first five F–T cycles owing to the gradual stabilization of the soil particle arrangement and interparticle bonding. Thereafter, the rate of strength loss decreased, suggesting that the geopolymeric matrix became more resistant to further deterioration. This behavior may stem from a more compact geopolymeric matrix that limits water ingress through pores and cracks, consequently reducing ice crystal formation during repeated F–T exposure, as reported by Min et al. [61].

The experimental results demonstrated distinct F–T responses between NSs stabilized with FA and NSs stabilized with FA-based geopolymer. For example, F30M0 exhibited a reduction in UCS of approximately 53% after 11 F–T cycles (from 506.30 to 236.30 kPa), whereas the optimum geopolymer-stabilized mixture, F30M8 maintained approximately 94% of its 28-day UCS. This superior durability may be associated with a compact geopolymeric framework containing possible C-(A)-S-H and/or N-A-S-H gels, which reduce the pore volume and enhance interparticle bonding [62,63]. Similarly, Tian et al. [64] reported that geopolymerization produced a more compact and uniform matrix containing fewer voids and microfractures, resulting in enhanced F–T resistance. Consequently, the reduced pore connectivity limits the volume of water susceptible to freezing within the treated soil, thereby reducing F–T-induced internal stresses and contributing to improved residual UCS after F–T cycles [65]. These findings indicate that 8 M NaOH provides optimum alkalinity for developing a durable geopolymeric matrix. A further rise in NaOH molarity to 10 M slightly reduced the durability performance, indicating that the optimum activator concentration had been exceeded. Excessive alkalinity may adversely affect geopolymerization, resulting in a less homogeneous geopolymeric matrix. Similarly, Adhikari et al. [66] reported that the concentration of NaOH and composition of the alkaline activator substantially influenced the strength development of FA-based soil geopolymers, highlighting the importance of optimizing activator systems.

**Figure 9:**
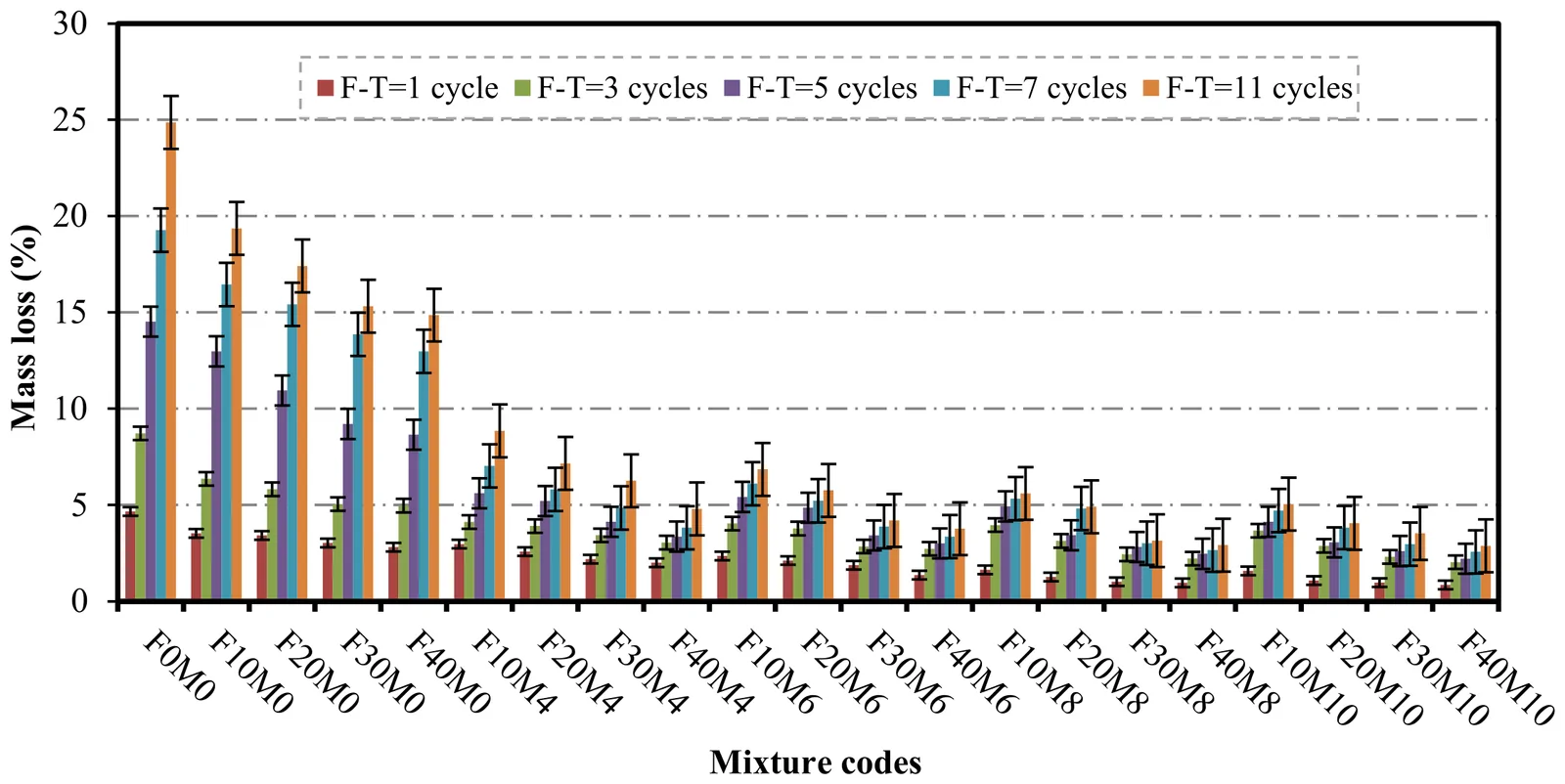
Effect of F–T cycles on the mass loss of all mixtures.

The resistance of the stabilized NSs was further assessed based on the mass loss after 1, 3, 5, 7 and 11 F–T cycles (Figure 9). Regardless of the mixture composition, the mass loss of all specimens increased progressively with an increasing number of F–T cycles. Similarly, Liu et al. [67] observed that mass loss accumulated as the F–T cycling continued. This trend indicates gradual surface deterioration and provides macroscopic evidence of structural deterioration [61]. Repeated F–T cycles progressively weaken the bond among soil particles and diminish the effectiveness of cementitious reaction products, thereby promoting microcrack development within the stabilized soil matrix [68,69]. The experimental results showed that the F0M0 mixture exhibited the poorest F–T durability, with a mass loss of 24.86% after 11 cycles. Among the NSs stabilized with FA, F30M0 exhibited improved F–T resistance compared to F0M0. However, it still experienced a substantial mass loss of 15.32% following 11 F–T cycles. This result suggests that the pozzolanic activity involving FA without alkaline activation was insufficient to provide adequate interparticle bonding against the stresses generated during repeated F–T cycles [70]. In contrast, FA-based geopolymer stabilization significantly enhanced the specimens’ resistance to F–T-induced damage (Figure 9). The optimum geopolymer mixture, F30M8, exhibited a remarkably low mass loss of only 3.15% after 11 F–T cycles, representing nearly a five-fold reduction compared with that of F30M0. This superior durability may be associated with the development of a compact and continuous geopolymeric framework containing possible C-(A)-S-H and/or N-A-S-H-type reaction products, which may contribute to the encapsulation of soil particles and the filling of microvoids [71]. Similarly, Chen et al. [72] reported that alkaline activation promoted gel formation, which encapsulated soil particles, filled pore spaces, and produced a denser matrix with improved F–T resistance. Likewise, Sun et al. [73] demonstrated that hydration products, including N-A-S-H and C-A-S-H gel phases, effectively formed bonds among the soil particles and filled pore spaces, resulting in a denser geopolymeric framework with greater resistance to F–T damage. Consistent with the UCS trends, mass loss increased most sharply over the early F–T cycles (1–5) but at a slower rate during the later cycles (7–11) (Figure 9). For instance, in the NSs stabilized with FA-based geopolymer, more than 75% of the total mass loss accumulated during the initial five cycles, suggesting that the geopolymeric matrix became progressively more resistant to further deterioration. This behavior agrees with Zhao et al. [59], who reported that F–T induced degradation was most severe during the initial cycles and progressed more gradually with continued cycling.

### 3.3. Sulfate Resistance of NSs Stabilized with FA-Based Geopolymer

**Figure 10:**
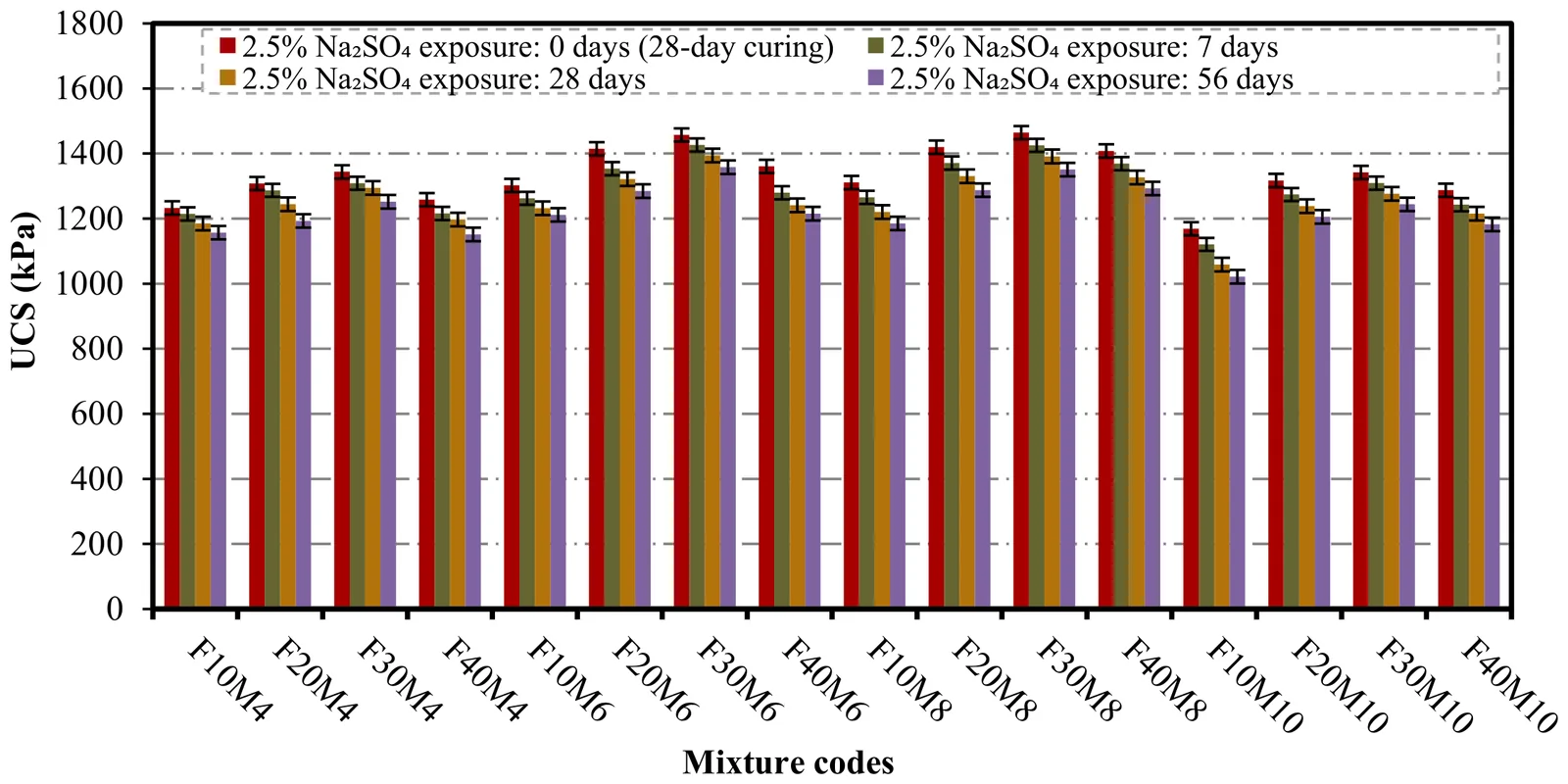
Effect of Na_2_SO_4_ exposure on the UCS of NSs stabilized with FA-based geopolymer.

**Figure 11:**
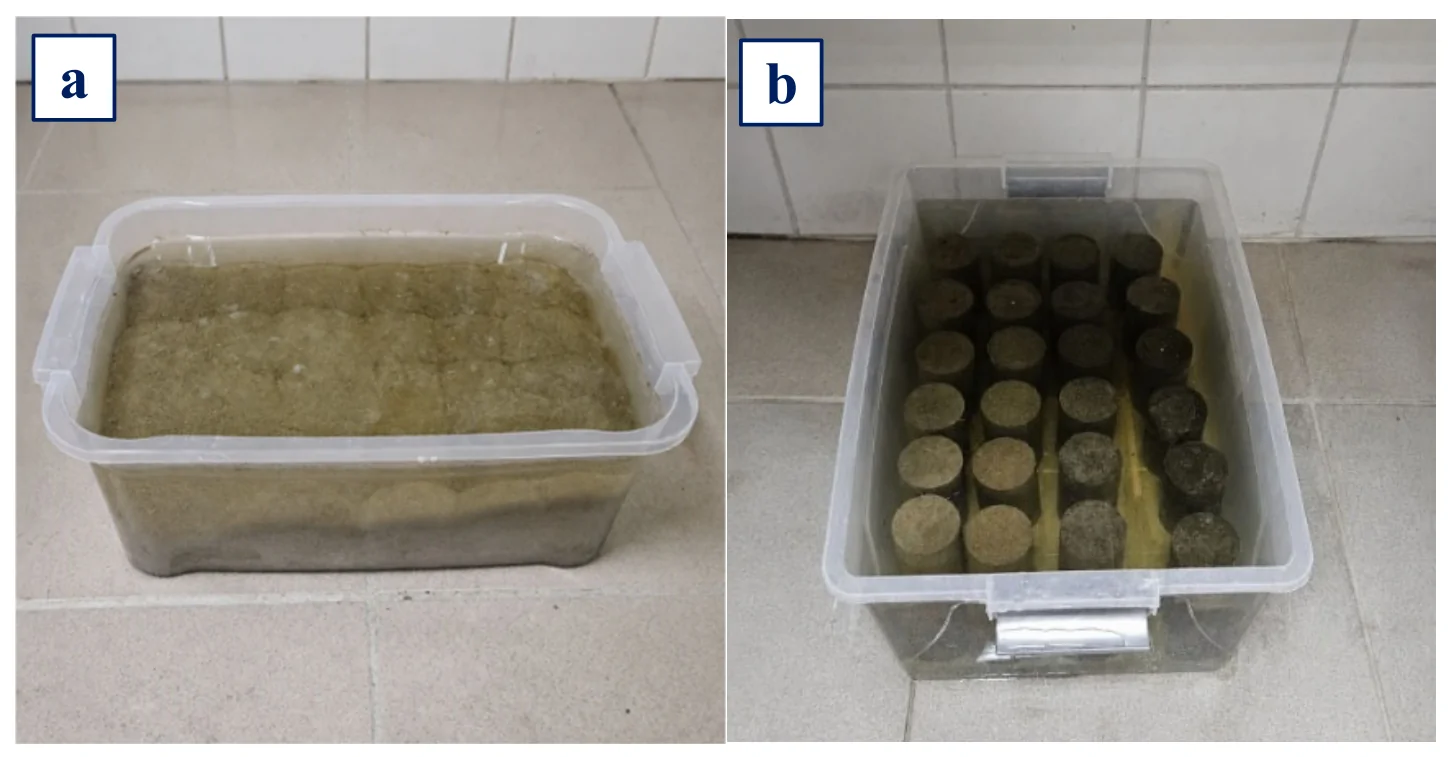
Sulfate-exposed specimens: (**a**) control; (**b**) NSs stabilized with FA-based geopolymer.

The changes in UCS for NSs stabilized with FA-based geopolymer following exposure to a 2.5% Na_2_SO_4_ solution for 7, 28 and 56 days are presented in Figure 10. All unactivated specimens, namely F0M0, F10M0, F20M0, F30M0, and F40M0, were observed to have completely disintegrated at the inspection conducted after 1 day of sulfate exposure (Figure 11a). Consequently, the scheduled UCS and mass-change measurements at 7, 28 and 56 days could not be performed for these specimens. These unavailable observations were treated as structurally missing durability-failure outcomes. They were neither assigned a value of zero nor statistically imputed and were therefore excluded from the corresponding quantitative plots and statistical analyses. Conversely, NSs stabilized with FA-based geopolymer maintained their structural integrity throughout the sulfate exposure period, indicating their enhanced resistance to sulfate attack (Figure 11b). The complete disintegration of F0M0 and NSs stabilized with FA demonstrated the damaging consequences of sulfate exposure. Within these matrices, sulfate ions may interact with calcium- and aluminum-bearing constituents, potentially contributing to the formation of expansive phases such as ettringite and thaumasite [74]. However, XRD and SEM-EDX analyses were not performed on the disintegrated specimens. Therefore, these phases are discussed only as possible literature-supported mechanisms rather than as confirmed causes of disintegration. If formed, such expansive phases could generate internal stress, promote cracking, and contribute to structural collapse [75]. In contrast, only the NSs stabilized with FA-based geopolymer remained visually and structurally intact throughout the sulfate exposure period, indicating comparatively improved durability under the applied laboratory conditions.

Furthermore, the NSs stabilized with FA-based geopolymer demonstrated high UCS retention under sulfate exposure. The optimum F30M8 mixture maintained approximately 92% of its initial 28-day UCS, decreasing from 1464.50 to 1351.01 kPa after 56 days of immersion. The measured UCS changes cannot be attributed exclusively to sulfate attack. Therefore, these changes are interpreted as net responses under the applied sulfate exposure conditions and may include the combined contributions of sulfate interaction, water immersion, and continued time-dependent reactions. The comparatively high UCS retention observed under the applied sulfate exposure conditions may be associated with the development of a compact and relatively stable alkali-activated framework containing possible C-(A)-S-H and/or N-A-S-H-type reaction products [76]. These possible reaction products may surround soil grains, occupy microvoids, and reinforce particle bonding [77]. The resulting compact matrix may have limited sulfate ingress into the stabilized soil, thereby mitigating sulfate-induced deterioration, cracking, and strength loss [78]. However, sulfate penetration depth, cross-sectional sulfur distribution, water absorption, permeability, and pore connectivity were not directly measured in this study. Therefore, the possible restriction of sulfate ingress is interpreted as an inference based on UCS retention, net mass change, visual integrity, and local microstructural observations rather than as a directly verified transport mechanism. This interpretation is consistent with previous studies reporting that a refined microstructure may reduce the penetration of sulfate ions (SO_4_^2−^) and limit the formation of expansive minerals such as ettringite and gypsum [79]. Consequently, the NSs stabilized with FA-based geopolymer exhibited significantly improved chemical durability and retained a high proportion of their initial UCS during sulfate exposure. Furthermore, the experimental results suggest that 8 M NaOH is the optimum activator concentration for developing a durable geopolymeric matrix (Figure 10). In contrast, raising the NaOH molarity to 10 M produced a greater decline in UCS, with the F10M10 mixture losing approximately 12.6% of its initial strength. Earlier research indicates that excessive alkalinity may increase the viscosity of the activating solution and promote the premature precipitation of aluminosilicate species, leading to the formation of a less homogeneous and more brittle geopolymeric matrix that is more susceptible to sulfate-induced microcracking [80].

**Figure 12:**
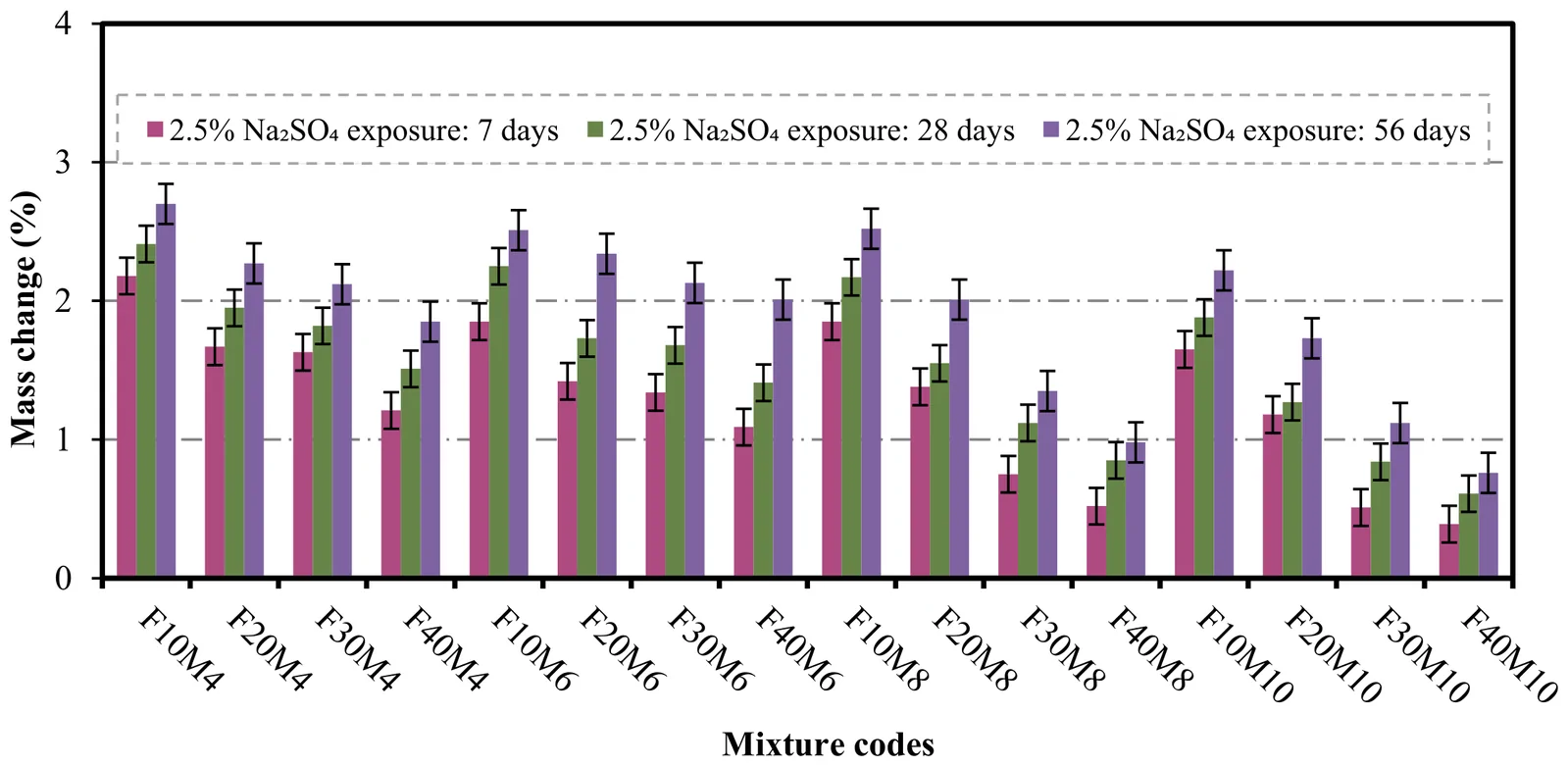
The mass change of NSs stabilized with FA-based geopolymer after sulfate exposure.

In addition to the UCS measurements, mass change was recorded throughout the immersion period to evaluate the physical response of the specimens under aggressive chemical conditions (Figure 12). The control specimens deteriorated rapidly during sulfate exposure, and subsequent mass measurements could not be performed (Figure 11a). Conversely, the NSs stabilized with FA-based geopolymer exhibited a slight and steady increase in mass (Figure 12). After 56 days of sulfate exposure, the optimum F30M8 exhibited a slight mass increase of 1.35%, whereas the lowest mass increase was recorded for the F40M10 specimen (0.76%). Despite exhibiting a lower UCS than the optimum F30M8, the reduced mass gain observed in the 10 M NaOH mixtures may be associated with excessive alkalinity. The high viscosity of the 10 M activator likely hindered the dissolution of silica and alumina and the development of geopolymeric reaction products, thereby affecting the internal pore structure and sulfate solution ingress [54]. Conversely, specimens containing lower FA contents and NaOH concentrations, such as F10M4, exhibited a greater mass increase, reaching 2.70%. Taken together, the observed values of 0.76–2.70% represent net mass increases and should not be interpreted as direct evidence of the formation of sulfate-related reaction products. Because the measurements were performed under SSD conditions rather than after drying to constant mass, the recorded values may include the combined effects of pore-water absorption, residual internal solution, salt crystallization, possible sulfate-related reactions, and material loss. According to previous studies, mass gain is primarily associated with the capillary absorption of the sulfate solution into the remaining micropores, followed by the crystallization of sulfate salts and/or the formation of secondary hydration products within the matrix [24,81]. Such processes may generate solid phases, including gypsum and secondary gels, from species dissolved in the solution, which may progressively fill the internal voids. Similarly, Jiang et al. [82] reported that the observed mass gain resulted from the reaction between sulfate ions and cementitious hydration products, including C-(A)-S-H and/or N-A-S-H and Ca(OH)_2_, leading to the formation of ettringite, thaumasite, gypsum, and water absorption into the pore structure. Likewise, Luo et al. [50] reported that the increase in specimen mass was governed by the transport of the Na_2_SO_4_ solution through capillary adsorption and water evaporation, with sulfate crystallization contributing to the gradual filling of the pore structure.

### 3.4. Microstructural and Mineralogical Characterization

The stabilization of NS using FA and FA-based geopolymer was investigated using SEM-EDX, XRD, and FTIR analyses to elucidate the molecular-level physical and chemical transformations associated with the stabilization process. The macroscopic changes observed in the UCS and durability against F–T and sulfate attack are directly associated with the microstructural and mineralogical evolution revealed by these analytical techniques. Microstructural and mineralogical characterizations were conducted on specimens after 28 days of standard curing, following exposure to 11 F–T cycles and after 28 days of sulfate exposure to evaluate the effects of these conditions on the stabilized specimens.

#### 3.4.1. SEM-EDX Analysis

**Figure 13:**
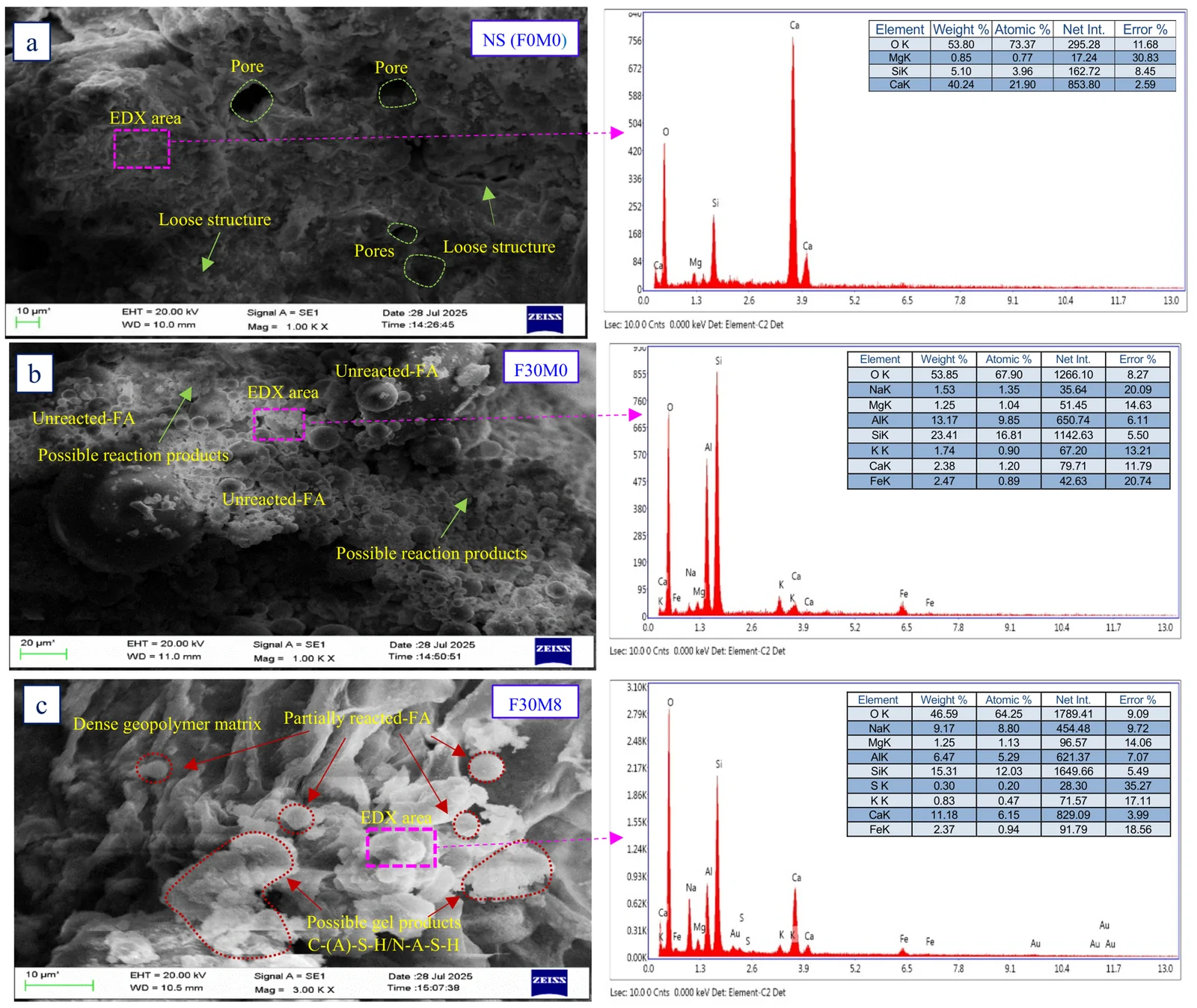
SEM-EDX analyses of (**a**) F0M0, (**b**) F30M0, and (**c**) F30M8 after 28 days of curing.

The SEM images and corresponding EDX spectra of F0M0, F30M0 and F30M8 after 28 days of curing are presented in Figure 13. F0M0 exhibited a loose and heterogeneous microstructure consisting of irregular clay particles with large, interconnected pores (Figure 13a). The loose particle arrangement and abundant pores indicate that the soil matrix remained largely unchanged after the curing process. The EDX spectrum of F0M0 was dominated by Ca and O, with moderate Si content and relatively low Mg content. Compared with F0M0, the incorporation of 30% FA transformed the loose and disordered microstructure into a denser and more integrated matrix (Figure 13b). This microstructural refinement may be attributed to the combined effects of the filler action of spherical FA particles, which occupy the intergranular voids, and possible chemical reactions during curing [49,83]. The EDX spectrum of F30M0 showed increased Si and Al contents, consistent with the incorporation of aluminosilicate-rich FA into the soil matrix. The observed elemental composition and microstructural refinement may reflect possible chemical reactions involving FA derived from silica and alumina during curing. The SEM image revealed numerous unreacted FA particles together with localized reaction products, which may be associated with possible chemical reactions occurring during curing [84]. Similarly, as reported by Raj et al. [85], while some FA particles remain inert fillers, others are coated with hydration products, particularly C-S-H, which contributes to the development of a denser microstructure and ultimately enhances UCS. In contrast to F0M0 and F30M0, F30M8 exhibited a markedly denser and more compact microstructure (Figure 13c). The reaction products appeared to form a more continuous and homogeneous binding matrix that effectively encapsulated the soil grains and reduced the interparticle voids, resulting in a well-integrated microstructure. A limited number of partially unreacted FA particles were observed, appearing to be embedded within and integrated into the surrounding reaction products rather than remaining as isolated particles, which is consistent with microstructural development under 8 M NaOH activation [14]. Similar observations were reported by Luo et al. [50], who identified unreacted FA particles together with C-(A)-S-H and/or N-A-S-H reaction products in FA-based geopolymer-stabilized soil using SEM analysis. The corresponding EDX spectrum further supports this morphological evolution by revealing a pronounced increase in the Na content along with considerable amounts of Si, Al, and Ca (Figure 13c). Similar findings were reported by Miraki et al. [86], who attributed the coexistence of Na, Si, Al, and Ca to the simultaneous formation of C-(A)-S-H and/or N-A-S-H gels, which contributed to the development of a denser and mechanically stronger matrix.

The detected Na is consistent with the incorporation of the alkaline activator. However, the EDX results alone do not directly demonstrate the dissolution of Si-O and Al-O bonds. As explained by Zhuang et al. [87], alkaline activation breaks the Si-O-Si and Si-O-Al bonds in the amorphous aluminosilicate phase of FA, releasing reactive Si and Al species that subsequently undergo polycondensation to form a geopolymeric network. The coexistence of Na, Si, Al, and Ca within the analyzed region is consistent with the possible formation of a complex binder system containing C-(A)-S-H and/or N-A-S-H-type reaction products [88]. These possible reaction products may have contributed to matrix densification through pore filling and improved interparticle bonding. Consequently, the resulting microstructural refinement may have reduced pore connectivity, enhanced soil-skeleton integrity, and contributed to the substantial increase in UCS observed for F30M8.

**Figure 14:**
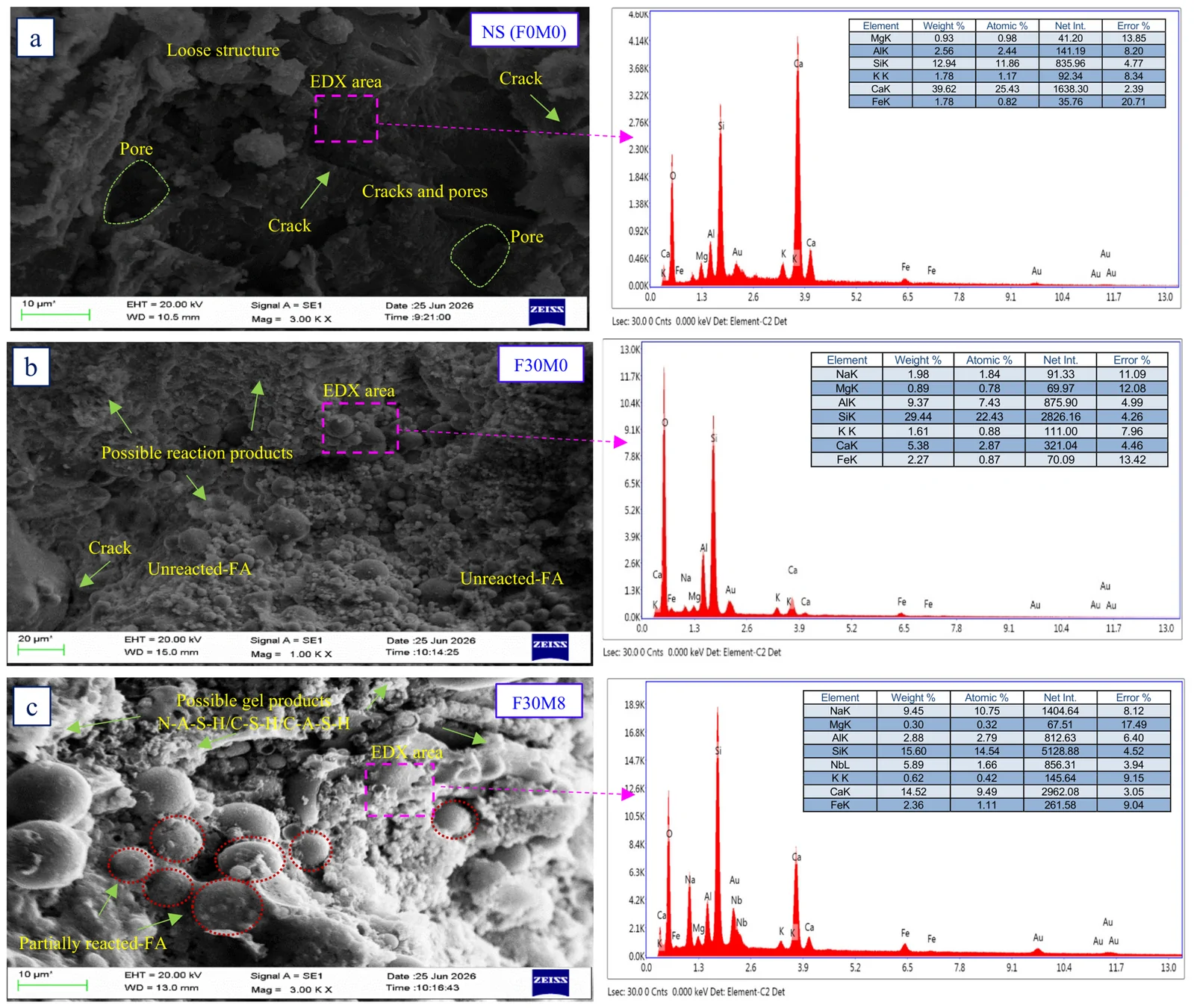
SEM-EDX analyses of (**a**) F0M0, (**b**) F30M0, and (**c**) F30M8 after 11 F–T cycles.

The SEM images and corresponding EDX spectra of F0M0, F30M0, and F30M8 after 11 F–T cycles are presented in Figure 14. F0M0 exhibited a severely deteriorated and discontinuous microstructure, characterized by extensive macropore development and weakening of interparticle bonds (Figure 14a). This deterioration is attributed to the internal expansion pressure generated by ice lens formation within the porous soil structure, which likely contributed to the significant reduction in UCS.

Compared with F0M0, F30M0 retained a relatively denser microstructure after 11 F–T cycles (Figure 14b). The EDX results were consistent with the possible preservation of calcium- and aluminosilicate-containing reaction products after F–T exposure, which may have contributed to mitigating structural deterioration. In contrast, F30M8 maintained a compact and well-integrated microstructure even after 11 F–T cycles (Figure 14c). As shown in the corresponding EDX spectrum, the coexistence of Na, Si, Al, and Ca is consistent with the possible preservation of C-(A)-S-H and/or N-A-S-H-type reaction products after F–T exposure. The possible preservation of these reaction products may have contributed to maintaining a relatively dense microstructure by strengthening interparticle bonding and limiting structural deterioration during F–T exposure [25]. Similarly, Dokaneh et al. [26] reported that these reaction products strengthened interparticle bonding by filling pore spaces, thereby maintaining a denser and more durable soil matrix during F–T exposure. This microstructural integrity contributed to the superior UCS, reduced mass loss, and enhanced durability of the NSs stabilized with FA-based geopolymer after F–T exposure.

**Figure 15:**
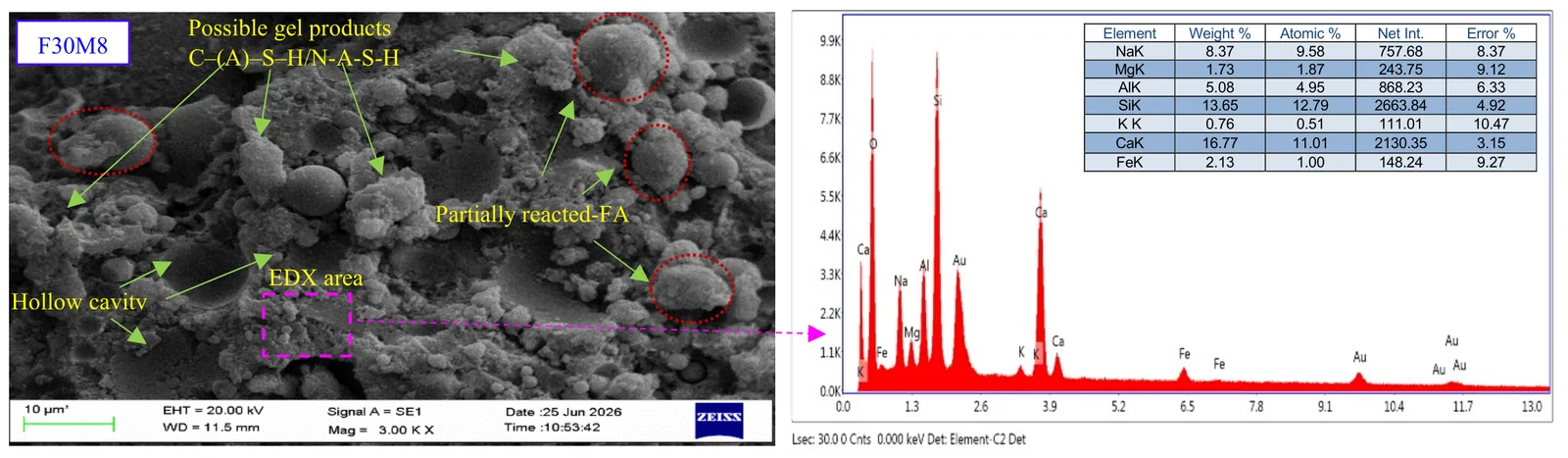
SEM-EDX analyses of F30M8 after 28 days of sulfate exposure.

The SEM image of the F30M8 specimen after 28 days of sulfate (Na_2_SO_4_) exposure is presented in Figure 15. F30M8 exhibited a dense and well-integrated microstructure, consistent with its superior sulfate durability. A dense and compact matrix was maintained, with possible alkali-activated reaction products distributed around the soil grains and the remaining FA particles. A distinctive morphological feature observed after sulfate immersion was the presence of circular imprints and hollow cavities within the matrix. These structures are consistent with alkaline erosion and the progressive dissolution of FA particles, during which reactive silica and alumina may have been released to sustain the geopolymerization process during sulfate exposure. Similarly, Cristelo et al. [89] attributed similar hollow cavities to the progressive dissolution of FA during alkaline activation, indicating that the cavities represented the former locations of partially dissolved FA particles. While some FA particles remained as partially reacted microfillers, most were incorporated into the binder system, indicating a high degree of reaction achieved with 8 M NaOH activation. The corresponding EDX spectrum provides information on the local elemental composition of the F30M8 matrix after sulfate exposure (Figure 15). The detected Na is consistent with the incorporation of the alkaline activator. The coexistence of Na, Ca, Si, and Al is consistent with the possible formation of C-(A)-S-H and/or N-A-S-H-type reaction products. The atomic Si/Al (≈2.58) and Ca/Si (≈0.86) ratios, calculated based on the elemental atomic percentages, are within the ranges reported in the literature for highly polymerized and dense binder systems [37,38]. However, these ratios alone do not permit the definitive identification of specific gel phases. The observed dense microstructure is consistent with the possibility of reduced sulfate ingress and may have contributed to maintaining structural continuity and preserving the relatively high UCS of the specimens. However, because SEM-EDX provides only local microstructural and elemental information, these observations do not directly confirm sulfate penetration or transport through the specimen.

#### 3.4.2. XRD Analysis

**Figure 16:**
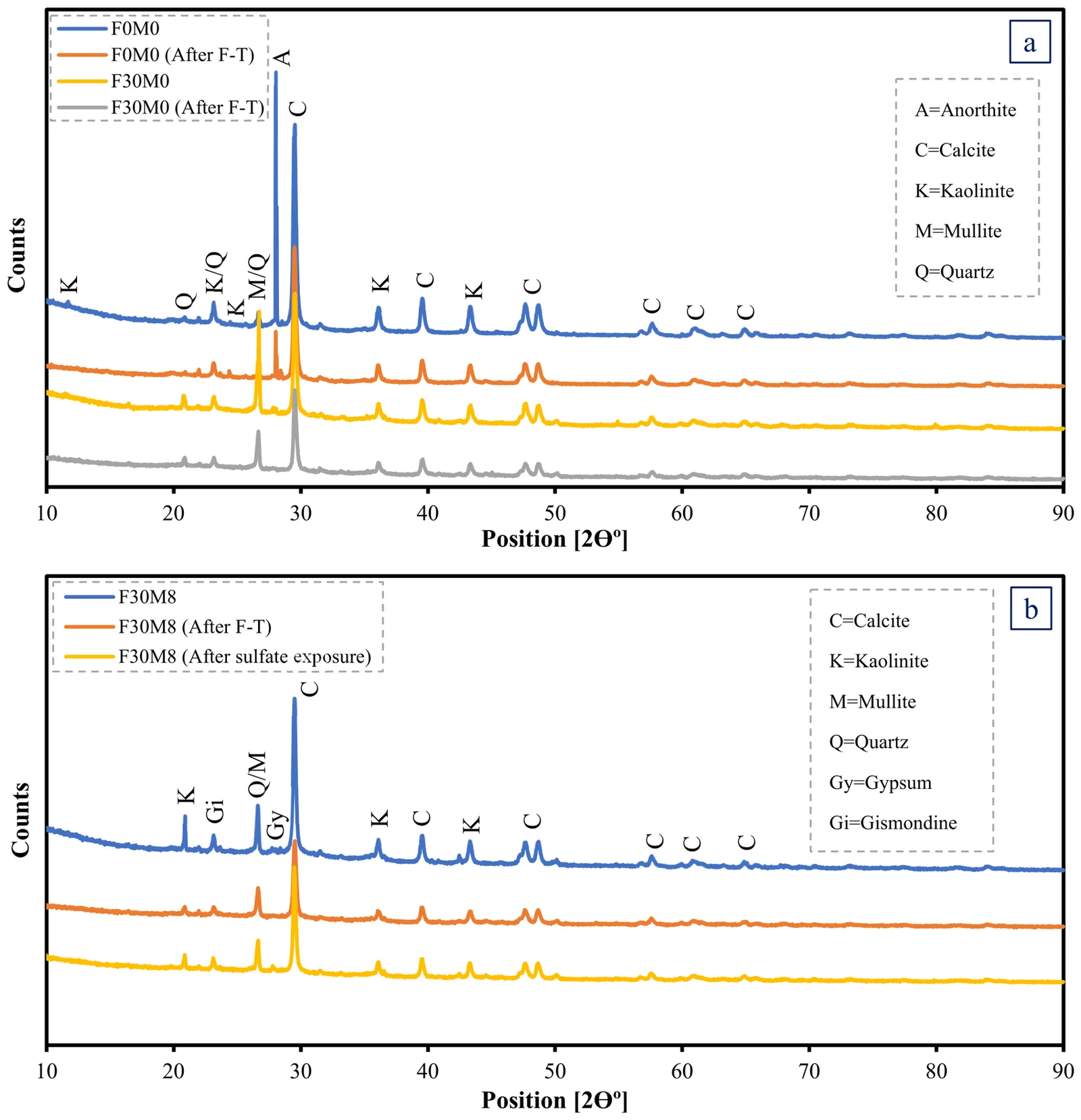
XRD patterns of (**a**) F0M0 and F30M0 specimens after curing and F–T cycles and (**b**) the F30M8 after curing, F–T cycles, and sulfate exposure.

The XRD patterns of the F0M0, F30M0, and F30M8 specimens after curing, 11 F–T cycles, and 28 days of sulfate exposure are presented in Figure 16. The diffraction pattern of F0M0 is predominantly characterized by quartz (Q) and calcite (C), accompanied by minor reflections of kaolinite (K) and mullite (M) and a weak anorthite (A). The incorporation of 30% FA (F30M0) resulted in a noticeable increase in the intensities of M and Q peaks. This observation is consistent with the presence of residual crystalline phases inherited from the FA precursor, which may primarily contribute through a microfiller effect within the soil matrix. Similar observations have been reported, indicating that unreacted FA particles remained within the matrix and mainly acted as physical fillers [90]. Compared with F0M0, F30M0 did not exhibit substantial changes in the crystalline assemblage, suggesting that the observed increase in UCS may be associated primarily with physical microfilling and the possible development of poor crystalline pozzolanic reaction products rather than newly formed crystalline phases [91] (Figure 16a). After 11 F–T cycles, the XRD pattern of F0M0 exhibited only limited variations, with no detectable formation of new crystalline phases. The slight relative reduction in peak intensities may be associated with physical changes in the soil fabric rather than a detectable mineralogical transformation. Accordingly, the mechanical deterioration observed in the unactivated mixture may primarily be related to physical structural damage caused by ice-crystal formation rather than chemical transformation [80]. Notably, because no internal standard or quantitative normalization procedure was used in the XRD measurements, the observed peak-intensity variations among the different patterns were interpreted only as qualitative mineralogical trends and relative crystalline changes and were not considered direct evidence of quantitative phase consumption. The incorporation of 8 M NaOH into the F30M8 mixture was accompanied by noticeable changes in its XRD pattern (Figure 16b). For F30M8, a pronounced amorphous hump appeared between 20° and 35° (2θ), which is consistent with the development of amorphous alkali-activated reaction products [88]. While this broad amorphous halo indicates the development of disordered geopolymeric and hydration structures, its confirmation is not made independently but is coupled with FTIR and SEM-EDX analyses to reliably support the formation of these gel phases. A distinct new reflection attributed to gismondine (Gi), a zeolite-type crystalline phase, emerged, which is consistent with mineralogical transformations associated with alkali activation [25]. This observation indicates the active transformation of aluminosilicate precursors into a more stable crystalline framework. Furthermore, a relative decrease in the intensity of the primary Q peaks was observed compared to that of F0M0. Because no internal standard or quantitative normalization procedure was used, this relative difference was not interpreted as direct evidence of quartz or silica consumption. Nevertheless, when considered together with the amorphous hump and the complementary FTIR and SEM-EDX observations, it may be consistent with the partial dissolution or structural reorganization of silica-bearing phases and the development of amorphous alkali-activated reaction products [55]. These mineralogical and microstructural changes may have contributed to the development of a denser binder network and, consequently, to the improved mechanical stability and environmental resistance observed in F30M8.

After 11 F–T cycles, the XRD pattern of F30M8 showed only a slight relative decrease in peak intensities, with no detectable formation of new crystalline phases, whereas sulfate exposure resulted in the emergence of gypsum (Gy) reflections. However, the persistence of the Gi phase and the relative stability of the alkali-activated matrix may have contributed to limiting structural degradation under these chemical and physical stressors (Figure 16b). Similarly, Liu et al. [67] reported that Gi was one of the main mineral phases, that the mineralogical composition remained largely unchanged throughout 10 F–T cycles, and that no new crystalline phases were formed.

#### 3.4.3. FTIR Analysis

**Figure 17:**
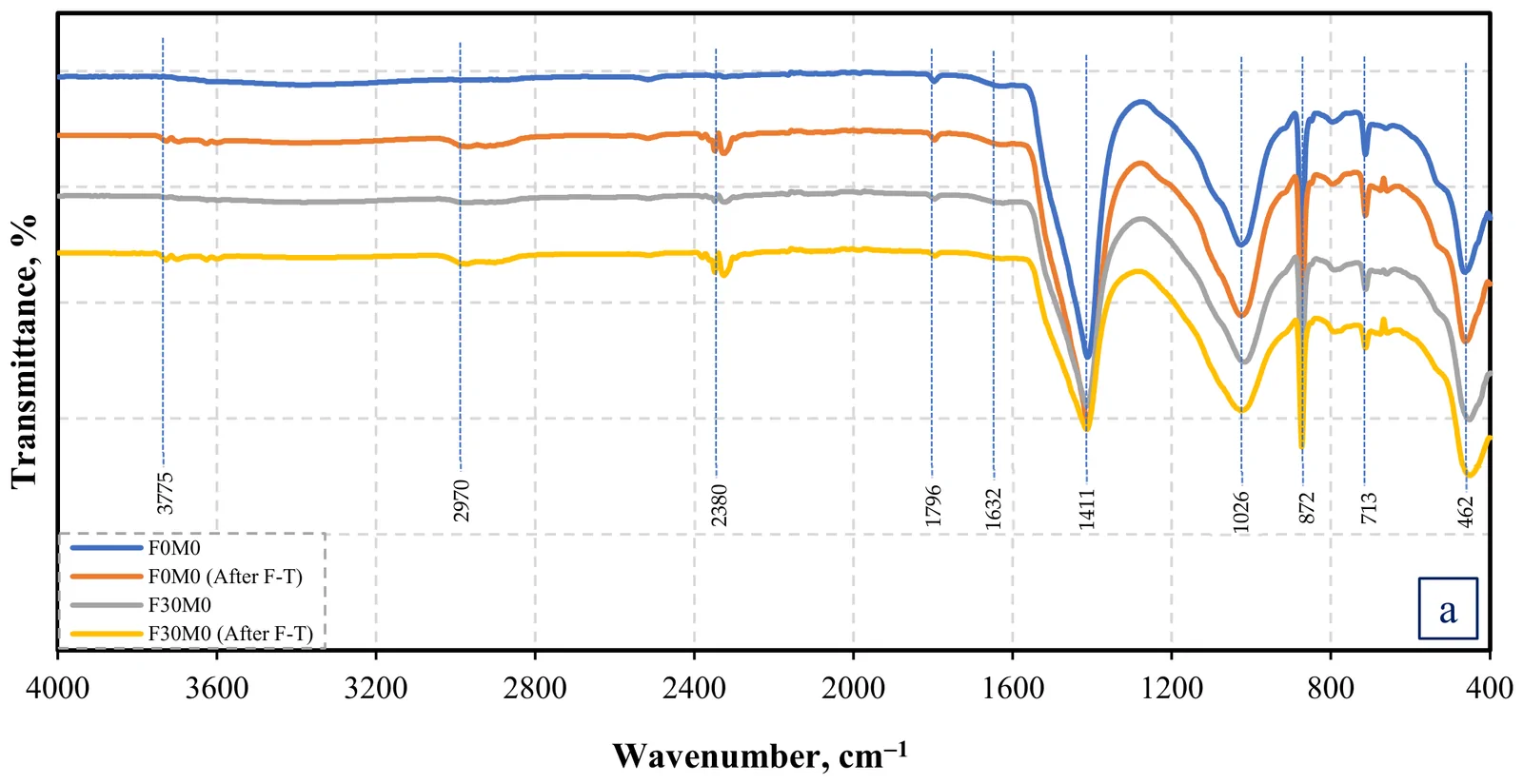

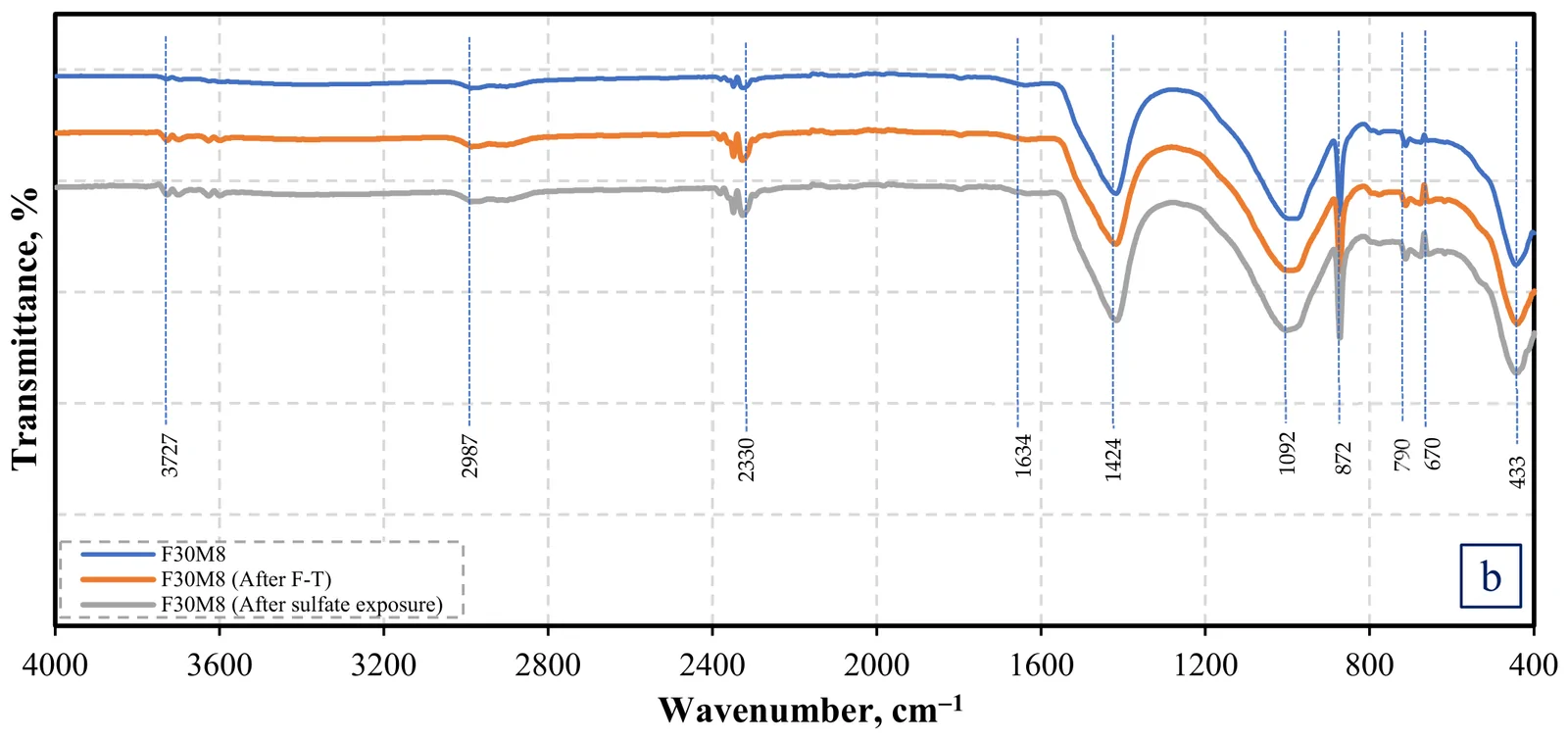
FTIR spectra of (**a**) F0M0 and F30M0 specimens after curing and F–T cycles, and (**b**) the F30M8 specimen after curing, F–T cycles, and sulfate exposure.

The FTIR results of the F0M0, F30M0 and F30M8 specimens after curing, 11 F–T cycles and 28 days of sulfate exposure are presented in Figure 17. The weak bands identified in samples F0M0 and F30M0 at approximately 3775 and 1632 cm^−1^ are attributed to O-H stretching and H-O-H bending vibrations, respectively. These bands indicate the presence of structural hydroxyl groups in clay minerals and physically bound water within the NS-FA matrix (Figure 17a). Similarly, Turkane and Chouksey [56] associated the bands at approximately 3432–3448 and 1634–1636 cm^−1^ with weakly bound water present as adsorbed water or within the voids of the reaction products. The minor bands observed at approximately 2970 and 2380 cm^−1^ are attributed to trace C-H stretching vibrations from organic impurities and atmospheric CO_2_ interference, respectively [92], and therefore were not regarded as evidence of the principal reaction products. The dominant absorption band at approximately 1026 cm^−1^ is attributed to the asymmetric stretching vibrations of Si-O-T (T = Si or Al) bonds [66], consistent with the crystalline quartz and clay minerals detected in the XRD patterns (Figure 16a).

Following alkaline activation, F30M8 exhibited a broad and intense Si-O-T band shifted to approximately 1092 cm^−1^ (Figure 17b), which is consistent with the dissolution and structural reorganization of aluminosilicate precursors and the development of an alkali-activated binder network. Consistent with this interpretation, Zhuang et al. [93] associated the shift of the Si-O-T band toward higher wavenumbers with progressive gel polymerization. The absorption bands at approximately 1424 and 872 cm^−1^ correspond to the stretching and bending vibrations of C-O bonds in carbonate groups, respectively, indicating limited carbonation during curing or sulfate exposure (Figure 17b) [60]. Furthermore, peaks identified at approximately 790, 670, and 433 cm^−1^ are primarily associated with Si-O and Si-O-T (T = Si or Al) vibrations in the residual crystalline phases (such as Q) and newly formed geopolymeric aluminosilicate framework [60,94]. Similar Si-O-Si stretching and bending vibrations have been reported at approximately 800 and 470 cm^−1^, respectively [95]. After 11 F–T cycles, the main absorption bands of F30M8 were preserved without the appearance of distinct new bands, indicating that the geopolymeric bonding structure remained stable. Similarly, the spectrum obtained after 28 days of sulfate exposure largely retained the main characteristic bands of the cured F30M8 specimen. The high degree of spectral consistency suggests that the geopolymeric framework largely maintained its chemical integrity and contributed to protecting the soil matrix against sulfate-induced degradation, despite the detection of Gy in the XRD pattern (Figure 16b).

### 3.5. Statistical Analysis Results

To systematically evaluate the statistical significance of the experimental parameters and their interaction effects on strength development, a three-way ANOVA was conducted on the UCS dataset (Table 5). The sulfate exposure dataset was not included in the three-way ANOVA because the complete disintegration of the unactivated specimens during sulfate exposure resulted in structurally missing observations that did not permit a balanced full-factorial analysis. These structurally missing observations represented durability-failure outcomes and were neither assigned a value of zero nor statistically imputed. Accordingly, ANOVA was restricted to the complete UCS dataset obtained at different curing ages, while the sulfate exposure results were reported as mean ± standard deviation. The ANOVA results revealed highly significant main effects of FA content, NaOH concentration, and curing age (*p* < 0.001). This confirmed that each parameter significantly influenced the UCS of the stabilized soil. All two-way interactions (FA × NaOH, FA × curing age, and NaOH × curing age) and the three-way interaction (FA × NaOH × curing age) were also highly significant (*p* < 0.001). In particular, the significant FA × NaOH interaction (F = 81.27, *p* < 0.001) indicates that the effect of NaOH concentration on UCS depends on the FA content. This finding supports the coupled influence of FA content and activator concentration on strength development, consistent with the interdependent reactions involved in geopolymerization.

To examine specific differences among the mixtures and support the selection of the best-performing mixture, a post-hoc Tukey’s HSD test was performed on the 56-day UCS results. The analysis showed that F30M8 (1488.58 kPa) exhibited significantly higher strength than F30M0 (528.88 kPa) and F0M0 (282.46 kPa) (*p* < 0.05). However, the difference between F30M8 and F30M10 was not statistically significant (*p* > 0.05). Accordingly, F30M8 was identified as the best-performing mixture among those investigated by considering its overall mechanical and durability performance together with the practical advantage of using a lower NaOH concentration than F30M10.

## 4. Conclusions

This study investigated the strength development and durability of a natural CH soil stabilized with Class F FA and FA-based geopolymer under curing, F–T cycles, and sulfate exposure. The principal outcomes derived from experimental testing and microstructural analyses are as follows:

- In the mixtures without alkaline activation, increasing the FA content to 30% improved the UCS. This improvement was primarily attributed to the microfiller effect of FA and possible time-dependent chemical reactions during curing. Raising the FA content to 40% decreased the UCS owing to the excessive presence of unreacted FA particles.
- Alkali activation resulted in substantially greater strength than the untreated NS and non-activated FA-stabilized specimens. F30M8, which contained 30% FA and was activated with 8 M NaOH, exhibited the highest UCS, reaching 1488.58 kPa after 56 days of curing, compared with 282.46 kPa for the untreated soil. This optimum mixture design, along with the highly significant main and interactive effects of all parameters, was statistically validated through a three-way ANOVA and post-hoc Tukey’s HSD tests (*p* < 0.05). Considering its overall mechanical and durability performance together with the practical advantage of using a lower NaOH concentration than F30M10, F30M8 was identified as the best-performing mixture among those investigated for the natural CH soil under the specified laboratory conditions.
- Repeated F–T cycles progressively reduced UCS and increased mass loss in all mixtures, with deterioration occurring most rapidly during the first five cycles. Nevertheless, F30M8 retained approximately 94% of its 28-day UCS and exhibited a mere 3.15% mass loss after 11 cycles. In comparison, F30M0 lost approximately 53% of its UCS and exhibited 15.32% mass loss, whereas F0M0 reached a mass loss of 24.86%.
- The F0M0 and NSs stabilized with FA rapidly deteriorated and eventually disintegrated during sulfate exposure. In contrast, NSs stabilized with FA-based geopolymer retained their structural integrity. F30M8 maintained approximately 92% of its initial 28-day UCS after 56 days of exposure to 2.5% Na_2_SO_4_ solution, demonstrating high resistance to sulfate attack.
- SEM-EDX observations revealed that F30M8 developed a dense, compact, and well-integrated microstructure, with the remaining FA particles embedded within a continuous binder matrix. The microstructural and chemical findings were consistent with the possible formation of C-(A)-S-H and/or N-A-S-H type reaction products under alkaline activation.
- XRD analysis revealed an amorphous hump between 20° and 35° (2θ), decreased Q peak intensities, and the formation of Gi in F30M8. FTIR analysis further indicated aluminosilicate restructuring through the shift of the main Si-O-T band from approximately 1026 to 1092 cm^−1^. The absence of major spectral changes following 11 F–T cycles and 28 days of sulfate exposure indicated that the geopolymeric bonding structure largely maintained its chemical integrity.
- While the laboratory results identify F30M8 as the best-performing mixture among those investigated in terms of mechanical and durability performance, its field-scale application requires careful management of challenges related to safety, logistics, equipment corrosion, and the cost of the 8 M NaOH activator. The use of appropriately adapted machinery, controlled activator-handling procedures, and field-scale economic assessments could enhance the practicality and applicability of this stabilization method.

The results indicate that the combination of 30% Class F FA and 8 M NaOH was the best-performing stabilization system among the mixtures investigated for the natural CH soil under the applied laboratory conditions. However, a limitation of this study is that F–T cycles and sulfate attack were evaluated separately rather than in combination, which represents a simplified approach to environmental weathering. Furthermore, because age-matched sealed-curing and deionized-water immersion controls were not included, the measured changes represent net responses under the applied exposure conditions and cannot be attributed exclusively to these environmental stressors. Therefore, because the findings are based on one natural soil and laboratory exposure conditions, further studies should examine different soil mineralogies, field-scale performance, and the combined/compounding effects of these environmental stressors to better understand soil behavior under compounding field conditions.

## Figures and Tables

**Table 3:** Mixture design and experimental conditions (specimen dimensions: 50 mm × 100 mm; number of replicates per mixture and test condition: *n* = 3).

Mix Code	FA (%)	Molar Concentration (M)	Number of F–T Cycles	Sulfate Exposure Duration (d)	Curing Time (d)
F0M0	0	0	1, 3, 5, 7, 11	7, 28, 56	1, 7, 28, 56
F10M0	10	0	1, 3, 5, 7, 11	7, 28, 56	1, 7, 28, 56
F10M4	10	4	1, 3, 5, 7, 11	7, 28, 56	1, 7, 28, 56
F10M6	10	6	1, 3, 5, 7, 11	7, 28, 56	1, 7, 28, 56
F10M8	10	8	1, 3, 5, 7, 11	7, 28, 56	1, 7, 28, 56
F10M10	10	10	1, 3, 5, 7, 11	7, 28, 56	1, 7, 28, 56
F20M0	20	0	1, 3, 5, 7, 11	7, 28, 56	1, 7, 28, 56
F20M4	20	4	1, 3, 5, 7, 11	7, 28, 56	1, 7, 28, 56
F20M6	20	6	1, 3, 5, 7, 11	7, 28, 56	1, 7, 28, 56
F20M8	20	8	1, 3, 5, 7, 11	7, 28, 56	1, 7, 28, 56
F20M10	20	10	1, 3, 5, 7, 11	7, 28, 56	1, 7, 28, 56
F30M0	30	0	1, 3, 5, 7, 11	7, 28, 56	1, 7, 28, 56
F30M4	30	4	1, 3, 5, 7, 11	7, 28, 56	1, 7, 28, 56
F30M6	30	6	1, 3, 5, 7, 11	7, 28, 56	1, 7, 28, 56
F30M8	30	8	1, 3, 5, 7, 11	7, 28, 56	1, 7, 28, 56
F30M10	30	10	1, 3, 5, 7, 11	7, 28, 56	1, 7, 28, 56
F40M0	40	0	1, 3, 5, 7, 11	7, 28, 56	1, 7, 28, 56
F40M4	40	4	1, 3, 5, 7, 11	7, 28, 56	1, 7, 28, 56
F40M6	40	6	1, 3, 5, 7, 11	7, 28, 56	1, 7, 28, 56
F40M8	40	8	1, 3, 5, 7, 11	7, 28, 56	1, 7, 28, 56
F40M10	40	10	1, 3, 5, 7, 11	7, 28, 56	1, 7, 28, 56

**Table 4:** Mass-based mixture proportions and compaction characteristics.

Mix Code	MDD (kN/m^3^)	OMC (%)	NaOH Molarity (M)	NS (g)	FA (g)	Water (g)	NaOH Pellets (g)
F0M0	12.45	35	0	400	0	140	0
F10M0	13.29	34	0	360	40	136	0
F10M4	13.29	34	4	360	40	136	21.76
F10M6	13.29	34	6	360	40	136	32.64
F10M8	13.29	34	8	360	40	136	43.52
F10M10	13.29	34	10	360	40	136	54.40
F20M0	13.36	33	0	320	80	132	0
F20M4	13.36	33	4	320	80	132	21.12
F20M6	13.36	33	6	320	80	132	31.68
F20M8	13.36	33	8	320	80	132	42.24
F20M10	13.36	33	10	320	80	132	52.80
F30M0	13.89	29	0	280	120	116	0
F30M4	13.89	29	4	280	120	116	18.56
F30M6	13.89	29	6	280	120	116	27.84
F30M8	13.89	29	8	280	120	116	37.12
F30M10	13.89	29	10	280	120	116	46.40
F40M0	13.96	26	0	240	160	104	0
F40M4	13.96	26	4	240	160	104	16.64
F40M6	13.96	26	6	240	160	104	24.96
F40M8	13.96	26	8	240	160	104	33.28
F40M10	13.96	26	10	240	160	104	41.60

**Table 5:** ANOVA results for the UCS of stabilized soil specimens.

Source of Variation	Sum of Squares (SS)	Degrees of Freedom (df)	Mean Square (MS)	F-Value	*p*-Value	Significance
FA Content	5.893 × 10^6^	3	1.964 × 10^6^	2828.85	<0.001	Highly Significant
NaOH Concentration	8.832 × 10^6^	4	2.208 × 10^6^	3179.92	<0.001	Highly Significant
Curing Age	8.975 × 10^6^	3	2.992 × 10^6^	4308.84	<0.001	Highly Significant
FA × NaOH	6.771 × 10^5^	12	5.643 × 10^4^	81.27	<0.001	Highly Significant
FA × Curing Age	1.455 × 10^5^	9	1.617 × 10^4^	23.28	<0.001	Highly Significant
NaOH × Curing Age	1.001 × 10^6^	12	8.340 × 10^4^	120.12	<0.001	Highly Significant
FA × NaOH × Age	2.259 × 10^5^	36	6.276 × 10^3^	9.04	<0.001	Highly Significant
Residual (Error)	1.111 × 10^5^	160	6.943 × 10^2^	-	-	-

## Data Availability

The original contributions presented in this study are included in the article. Further inquiries can be directed to the corresponding author.
